# Immune microenvironment modulation unmasks therapeutic benefit of radiotherapy and checkpoint inhibition

**DOI:** 10.1186/s40425-019-0698-6

**Published:** 2019-08-13

**Authors:** Jared M. Newton, Aurelie Hanoteau, Hsuan-Chen Liu, Angelina Gaspero, Falguni Parikh, Robyn D. Gartrell-Corrado, Thomas D. Hart, Damya Laoui, Jo A. Van Ginderachter, Neeraja Dharmaraj, William C. Spanos, Yvonne Saenger, Simon Young, Andrew G. Sikora

**Affiliations:** 1 Department of Otolaryngology-Head and Neck Surgery, Baylor College of Medicine, Houston, TX USA; 2 Interdepartmental Program in Translational Biology and Molecular Medicine, Houston, TX USA; 3 Department of Pediatrics, Division of Pediatric Hematology/Oncology, Columbia University Irving Medical Center/New York Presbyterian, New York, NY USA; 4 Department of Medicine, Division of Hematology/Oncology, Columbia University Irving Medical Center/New York Presbyterian, New York, NY USA; 5 Laboratory of Cellular and Molecular Immunology, Vrije Universiteit Brussel (VUB), Brussels, Belgium; 6 Laboratory of Myeloid Cell Immunology, VIB Center for Inflammation Research, Brussels, Belgium; 7 Department of Oral and Maxillofacial Surgery, School of Dentistry, The University of Texas Health Science Center at Houston, Houston, TX USA; 8 Department of Surgery, University of South Dakota, Sanford School of Medicine, Vermillion, SD USA; 9 Department of Cell and Gene Therapy, Baylor College of Medicine, Houston, TX USA

**Keywords:** Immunotherapy, Tumor immune microenvironment, Immune checkpoint inhibitors, Programmed cell death protein-1 (PD-1), Cytotoxic T lymphocyte associated antigen-4 (CTLA-4), Cyclophosphamide (CTX), L-n6-(1-iminoethyl)-lysine (L-NIL), Radiotherapy, Head and neck cancer, Human papillomavirus (HPV)

## Abstract

**Background:**

Immune checkpoint inhibitors (ICIs) for solid tumors, including those targeting programmed cell death 1 (PD-1) and cytotoxic T lymphocyte-associated antigen 4 (CTLA-4), have shown impressive clinical efficacy, however, most patients do not achieve durable responses. One major therapeutic obstacle is the immunosuppressive tumor immune microenvironment (TIME). Thus, we hypothesized that a strategy combining tumor-directed radiation with TIME immunomodulation could improve ICI response rates in established solid tumors.

**Methods:**

Using a syngeneic mouse model of human papillomavirus (HPV)-associated head and neck cancer, mEER, we developed a maximally effective regimen combining PD-1 and CTLA-4 inhibition, tumor-directed radiation, and two existing immunomodulatory drugs: cyclophosphamide (CTX) and a small-molecule inducible nitric oxide synthase (iNOS) inhibitor, L-n6-(1-iminoethyl)-lysine (L-NIL). We compared the effects of the various combinations of this regimen on tumor growth, overall survival, establishment of immunologic memory, and immunologic changes with flow cytometry and quantitative multiplex immunofluorescence.

**Results:**

We found PD-1 and CTLA-4 blockade, and radiotherapy alone or in combination, incapable of clearing established tumors or reversing the unfavorable balance of effector to suppressor cells in the TIME. However, modulation of the TIME with cyclophosphamide (CTX) and L-NIL in combination with dual checkpoint inhibition and radiation led to rejection of over 70% of established mEER tumors and doubled median survival in the B16 melanoma model. Anti-tumor activity was CD8^+^ T cell-dependent and led to development of immunologic memory against tumor-associated HPV antigens. Immune profiling revealed that CTX/L-NIL induced remodeling of myeloid cell populations in the TIME and tumor-draining lymph node and drove subsequent activation and intratumoral infiltration of CD8^+^ effector T cells.

**Conclusions:**

Overall, this study demonstrates that modulation of the immunosuppressive TIME is required to unlock the benefits of ICIs and radiotherapy to induce immunologic rejection of treatment-refractory established solid tumors.

**Electronic supplementary material:**

The online version of this article (10.1186/s40425-019-0698-6) contains supplementary material, which is available to authorized users.

## Background

Solid tumors currently account for over 90% of new cancer cases and cancer-related deaths in the U. S [1]. Alongside conventional treatments such as chemotherapy, radiotherapy, and surgery, immunotherapy has recently emerged as a standard of care treatment for diverse recurrent/metastatic tumors. Among cancer immunotherapies, immune checkpoint inhibitors (ICIs) describe a class of drugs which block proteins that downregulate immune responses. In 2011 the first ICI, a monoclonal antibody targeting cytotoxic T lymphocyte-associated protein 4 (anti-CTLA-4 or αCTLA-4), was approved for use in advanced melanoma and followed in 2014 by another ICI targeting programmed cell death protein 1 (anti-PD-1 or αPD-1) [2–5]. Both αCTLA-4 and αPD-1 are currently clinically approved or under investigation for use in numerous solid tumor malignancies [6]. Although some patients achieve long-term, seemingly curative, responses to ICI monotherapies, approximately 60–80% of patients do not receive durable benefit from these therapies [7–9]. In an effort to potentiate the therapeutic efficacy of ICIs various combinatory approaches have been investigated, including dual ICI approaches [10–12] and combinations with standard-of-care therapies (i.e. chemotherapy and radiation) as well as other immunotherapies [13, 14]. Tumor-directed radiation, in particular, has shown promising combinatorial benefit with ICIs, driven largely by its ability to stimulate tumor cell apoptosis and antigen uptake [15]; increase the expression of major histocompatibility complex class I (MHCI) on cancer cells [16]; and promote tumor-specific clonal T cell focusing [13, 17, 18]. However, radiation also promotes substantial lymphodepletion [19, 20] and immunosuppressive effects, including impaired T cell reactivity; diminished antigen presentation; and elevation of circulating immunosuppressive cells [21, 22]. This suggests that additional therapeutic combinations may be required to unmask the maximum benefit of ICIs.

Recent advances in our understanding of the tumor-immune interaction suggest that effective anti-tumor immunity requires a complex and multi-faceted response. This includes: (i) promotion of immunogenic tumor cell death and antigen release, (ii) antigen uptake and effective presentation by antigen presenting cells (APCs), (iii) generation and priming of tumor-specific cytotoxic T cells, (iv) migration and infiltration of those T cells into the tumor environment, and (v) continuous T cell recognition and killing of tumor until clearance [23, 24]. This poses a challenge to current cancer immunotherapies, since most immunomodulators are only capable of stimulating a few of the necessary steps listed above when used as a single agent. An equally daunting challenge is the highly immunosuppressive tumor immune microenvironment (TIME). As a recently recognized hallmark of solid tumor cancers [25], the TIME is often characterized by the infiltration of various immunosuppressive cell types, most notably myeloid-derived suppressor cells (MDSCs) and regulatory T cells (Tregs), and a lack of anti-tumor immune activity (often described as a “cold” tumor) [26, 27]. Numerous studies have demonstrated the profound effects that the TIME can have on treatment response, not just for immunotherapies, but for numerous oncologic modalities [27–29]. Thus, favorably remodeling the TIME could sensitize tumors to ICI therapy benefit; however, there are currently few clinically available immunomodulatory strategies capable of broadly reprogramming the various myeloid and lymphoid cellular subsets comprising the TIME.

Our group has previously shown that the combination of cyclophosphamide (CTX) and a selective small molecule inducible nitric oxide synthase (iNOS) inhibitor, L-n6-(1-iminoethyl)-lysine (L-NIL), provides potent intratumoral immunomodulatory effects. More specifically, we demonstrated that L-NIL inhibits MDSC development and intratumoral trafficking [30], and when combined with CTX prevents Treg tumor infiltration [31]. Mitigation of these two immunosuppressive cells using CTX and L-NIL (CTX/L-NIL) ultimately promoted enhanced infiltration of CD8^+^ T cells and improved survival in a mouse model of melanoma [31]. In an additional murine model of human papillomavirus (HPV) head and neck squamous cell carcinoma (HPV-HNSCC) we observed that CTX/L-NIL promotes even broader immunologic effects, including the upregulation of numerous anti-tumoral immune pathways such as antigen processing and presentation, myeloid trafficking and activation, and T cell function and co-stimulation [32]. We further found that its combination with chemoradiotherapy promoted favorable alterations in both the myeloid and lymphoid intratumoral microenvironment which significantly enhanced the therapeutic benefit of standard-of-care therapy [32]. Thus, we hypothesized that CTX/L-NIL immunomodulation could promote a “cold to hot” transition of the TIME which could enhance treatment responses to ICI and radiation therapies.

To test this hypothesis, we used a syngeneic model of HPV-HNSCC (mEER) developed using murine pharyngeal epithelial cells transduced with HPV16 E6 and E7 viral oncogenes and H-ras [33, 34] with additional validation in models of HPV negative HNSCC and melanoma. We observed that established mEER tumors minimally respond to ICI therapies and suggest this to be due to their inability to overcome the immunosuppressive TIME. When ICIs are combined with radiation, though therapeutic benefit is improved, they remain non-curative and the TIME remains “cold”, with low effector-to-suppressor immune infiltrate. However, when ICIs and radiation are combined with CTX/L-NIL immunomodulation, the combination induces complete regression and clearance of over 70% of established tumors in a CD8^+^ T cell-dependent manner, accompanied by establishment of potent tumor-antigen specific memory. This dramatic improvement in treatment efficacy is attributed to broadly favorable alterations of the TIME and tumor-draining lymph node (tdLN). More specifically we observed (i) beneficial alterations of tumor-infiltrating myeloid cells at early treatment timepoints, which lead to (ii) enhanced tdLN infiltration of anti-tumor myeloid cells and T cell proliferation, which ultimately drives (iii) enhanced intratumoral T cell infiltration and activation at later treatment timepoints. Overall, these findings implicate the TIME as a major barrier to ICI therapies and demonstrate that its effective modulation can unlock their therapeutic potential in solid tumors.

## Methods

### Experimental design

The primary objectives of this study were (i) to identify tumor features that limit immune checkpoint inhibitors therapeutic benefit in established solid tumors and (ii) to develop combinatory treatment strategies to maximize their efficacy. All experiments were replicated at least two times with an average of 5–10 samples per experiment, and final representation includes either pooled data or representative experiments, as noted in the corresponding figure legends. The number of mice used per experiment was determined using power analysis (α = 5%, β = 20%) and prior knowledge of experimental variability. The precise number of mice used within each experiment is presented in the corresponding figure legend. To limit cage-specific effects, mice were randomized across experimental groups prior to treatment initiation. All measurements were recorded under proper treatment blinding. Outliers from flow cytometry analysis were determined using the ROUT (Q = 1%) method and excluded from the analysis.

### Mice

C57BL/6 J male mice were purchased from The Jackson Laboratory and housed under specific pathogen-free conditions in standard temperature and lighting conditions with free access to food and water. Tumor inoculation was performed when mice reached 8–10 weeks of age. All experiments were performed with approval of the Institutional Animal Care and Use Committee (IACUC) at Baylor College of Medicine (BCM) and followed established protocols.

### Tumor model

mEER cell line expressing HPV16 E6, E7 and hRas was obtained from Dr. John Lee at the Sanford Research center/ University of South Dakota and maintained in E-media as previously described [33]. MOC2 cell line was obtained from Dr. Uppaluri at Brigham and Women’s Hospital/ Harvard Medical School and maintained as previously described [35, 36]. MOC2 E6/E7 cell line expressing HPV16 E6 and E7 was obtained from Dr. Simon Young at UT Health and was maintained similar to the parental MOC2 cell line [37]. B16-F0 cell line was purchased from American Type Culture Collection (ATCC) and maintained according to manufacturer instructions (DMEM high-glucose with 10% fetal bovine serum and 1% penicillin/streptomycin). C57BL/6 J mice were injected subcutaneously (s.c.) with 1 × 10^6^ mEER, 1 × 10^5^ MOC2, 1 × 10^5^ MOC2 E6/E7, or 3 × 10^5^ B16-F0 cells in the flank. Mice were monitored 2–3 times per week for tumor growth using calipers. Tumor area (mm^2^) was calculated as L x W, where L is Length and W is Width, respectively. Growth curve experiments were stopped once tumors reached 225 mm^2^.

### In vivo treatment

All mice were properly randomized prior to treatment. Once tumors become established (day 17–18 after tumor inoculation for mEER tumors and day 4 for B16-F0 tumors) treatment was initiated. Mice then received combinations of treatment including immune checkpoint inhibitors, tumor directed radiation, and/or CTX/L-NIL immunomodulation. Immune checkpoint inhibitors, *InVivo*MAb anti-mouse PD-1 (clone RMP1–14; BioXCell; 250 μg per dose) and/or *InVivo*MAb anti-mouse CTLA-4 (clone 9H10; BioXCell; 100 μg per dose), were administered using intraperitoneal (i.p.) injections for a total of 6 doses. Control mice received combination isotype antibodies to account for non-specific antibody effects; *InVivo*MAb rat IgG2a (clone 2A3; BioXCell; 250 μg per dose) and/or *InVivo*MAb Syrian Hamster IgG (polyclonal; BioXCell; 100 μg per dose). Tumor-directed radiation was delivered as a 2 X 10 Gy regimen (each dose delivered weekly). Irradiation was performed on non-anesthetized mice using a RadSource 2000 X-ray irradiator (160 kV, 25 mA) at a dose rate of 0.031 Gy/s. Each mouse was briefly confined in a plastic restrainer and tumor-directed radiation was done using lead shield with an opening that exposed the tumor-bearing flank of the mouse (BrainTree Scientific, Inc.). The immunomodulatory regimen was delivered over 2 weeks and combined a weekly cyclophosphamide (2 mg/mouse; TCI Chemicals) i.p. injection with continuous L-NIL (2 mg/mL; Enzo Life Sciences) in the drinking water (see Fig. 2b for treatment schematic).

For CD8 depletion experiments, all mice receiving the full treatment regimen were injected with 1 mg depleting *InVivo*MAb anti-mouse CD8α (clone 53–6.7; BioXCell) or *InVivo*MAb rat IgG2a isotype control (clone 2A3; BioXCell) 2 days prior the treatment, and further treated with 250 μg of depleting antibody weekly for 4 consecutive weeks (see Fig. 6a for treatment schematic).

### Gene expression analysis

Tumor samples were harvested and flash frozen in liquid nitrogen. Total RNA was extracted with the RNeasy Mini Kit (Qiagen) as per the manufacturer’s instructions. Gene expression profiling was performed on 100 ng RNA using the nCounter® PanCancer Immune Profiling Panel (NanoString Technologies, Inc) containing 770 genes involved in cancer immune response. Gene expression profiling was performed using the NanoString nCounter® Gene Expression system. The process including the following steps: (i) Hybridization protocol: 100 ng of total RNA were hybridized with the NanoString Technologies nCounter® Gene Expression Mouse PanCancer Immune Profiling code set containing 770 unique pairs of 35-50 bp reporter probes and biotin-labeled capture probes, including internal reference controls. Overnight hybridization occurred for 17–22 h at 65 °C. (ii) Wash protocol: Removal of excess probes with magnetic bead purification was performed on the nCounter® Prep Station (software v4.0.11.2) on the High Sensitivity assay. Briefly, the probe-mRNA structure was affinity purified by its 3′ end to remove excess reporter probes, then by its 5′ end to remove excess capture probes. Once unbound probes were washed away, the tripartite structure was bound to the streptavidin-coated cartridge by the biotin capture probe, aligned by an electric current (negative to positive), and immobilized. Photobleaching and fluorophore degradation was prevented with the addition of SlowFade. (iii) Scan protocol: The cartridge containing immobilized samples was transferred to the nCounter® Digital Analyzer (software v3.0.1.4) and scanned at 555 field of view (FOV). An epi-fluorescent microscope and CCD camera identified sets of fluorescent spots, which were tabulated for data output. Quality control metrics were recorded using the nSolver Analysis Software v3.0.22. Raw read counts were normalized, background subtracted, and assessed for cell type score and differential gene expression using NanoString nSolver (version 3.0) following the manufacture instruction.

### Flow cytometry assessment of immune microenvironment

To observe tumor immune cell infiltration, mEER tumors were harvested, digested and stained using the method previously describe [38]. Briefly, tumors were digested in RPMI 1640 (Sigma-Aldrich) containing DNase I (20 U/ml; Sigma-Aldrich), Collagenase I (1 mg/ml; EMD Millipore) and Collagenase IV (250 U/ml; Worthington Biochemical Corporation) prior to mechanical disaggregation to form single cell suspensions. Following digestion, tumor infiltrating leukocytes were enriched using Lymphoprep™ (STEMCELL Technologies). Single cell suspensions were also prepared from tumor-draining inguinal lymph node and spleen with additional lysis of splenic red blood cells (RBC) using RBC lysis buffer (Invitrogen). For extracellular staining, all cells were first blocked with anti-mouse CD16/CD32 Fc block (BD Biosciences) and separately stained using one of various antibody panels (see Additional file 14: Table S1 for antibody panels). E7 MHCI tetramer with conjugated BV421 was used for E7-specific CD8^+^ T cell staining (NIH Tetramer Core Facility). For intracellular staining, cells were fixed and permeabilized with Intracellular Fixation and Permeabilization Buffer Set (eBioscience) prior to the addition of intracellular staining antibody sets. Data were acquired on a LSRII and LSRFortessa (BD Biosciences) flow cytometers, for myeloid and T cell panels respectively, and analyzed using FlowJo v10 software (FlowJo, LLC). Cellular or cellular phenotype percentage changes were often converted to Z-scores by taking the entire dataset average and standard deviation and then calculating how many dataset standard deviations a given sample was away from that population average. In some cases all single sample Z-scores for a given treatment were averaged together to give an average treatment Z-score.

### Quantitative multiplex immunofluorescence

#### Sectioning and staining

After harvesting, tumors were immediately fixed overnight in 10% neutral-buffered formalin. Fixed tumors were dehydrated using an ethanol series, embedded in paraffin, and sections were cut at a thickness of 5 μm. Full-section slides of tumor tissues were stained using Opal multiplex 6-plex kits, according to the manufacturer’s protocol (Akoya), for DAPI, Epcam (polyclonal; Abcam, 1:100 dilution), CD3 (clone SP7; Spring Biosciences; 1:100 dilution), CD8 (clone 4SM15; Thermo Fisher; 1:500), CD4 (clone 4SM95; eBioscience, 1:50), Foxp3 (polyclonal; Thermo Fisher, 1:500), and Granzyme B (polyclonal; Abcam, 1:200). Single color controls and an unstained slide were also included for proper spectral un-mixing.

#### Multispectral imaging

Multispectral image capture was done at 20X magnification using Vectra (Akoya). Images were analyzed using inForm software version 2.4.1 (Akoya) as previously described [39]. Briefly, five representative areas were randomly selected. These images were factored equally into the analysis for each mouse. For spectral un-mixing, examples of each fluorophore are taken from single-stained slides for each antibody, as well as a representative autofluorescence spectrum from an unstained sample.

#### Automated analysis

Images from each of these single-stained and unstained slides were used to create a multispectral library in inForm and extracted from the multispectral data using linear un-mixing. Cellular and subcellular compartments were defined by a counterstain (DAPI) to define the nucleus of each cell. Cell segmentation was adjusted based on minimum DAPI signal to accurately locate all cells and minimize hyper- and hypo-segmentation below 5% of total cells (assessed manually). Cells were then characterized using the phenotyping feature in inForm. Approximately 25–30 representative cells for each base variable were selected to train the phenotyping algorithm: tumor (EpCAM), T cells (CD3), and other (negative for EpCAM and CD3). Last the images were scored for intensity based on each individual secondary marker for further phenotyping of CD4, CD8, FoxP3, and Granzyme B. Finally, data obtained from all representative images were compiled to yield values for each mouse. Exported inForm data from all images were processed in separate software designed in RStudio (version 0.99.896). In this software, images were combined and analyzed to concatenate variables (i.e., CD3^+^CD8^+^Granzyme B^+^) and determine density and distance of distinct phenotypes. Densities were all calculated as counts per total nucleated cells.

### Statistical analysis

Data sets were tested for Gaussian distribution using the D’Agostino-Pearson normality test. For parametric data sets, statistical significance was determined by: unpaired t test for two-tailed data or ANOVA test followed by selected comparison using Tukey’s multiple comparison tests with multiple comparison correction. For non-parametric data sets, statistical significance was determined by: Mann-Whitney test for two tailed data and Kruskal-Wallis test followed by selected comparison by Dunn’s multiple comparison tests with multiple comparison correction. Survival was analyzed by the Kaplan– Meier method using Log-rank test. (*p < 0.05; **p < 0.01; ***p < 0.001; ****p < 0.0001; ns, non-significant). Outliers from flow cytometry analysis were determined using ROUT (Q = 1%) method.

## Results

### Immune checkpoint inhibitors alone and in combination weakly inhibit mEER tumor growth

**Fig. 1:**
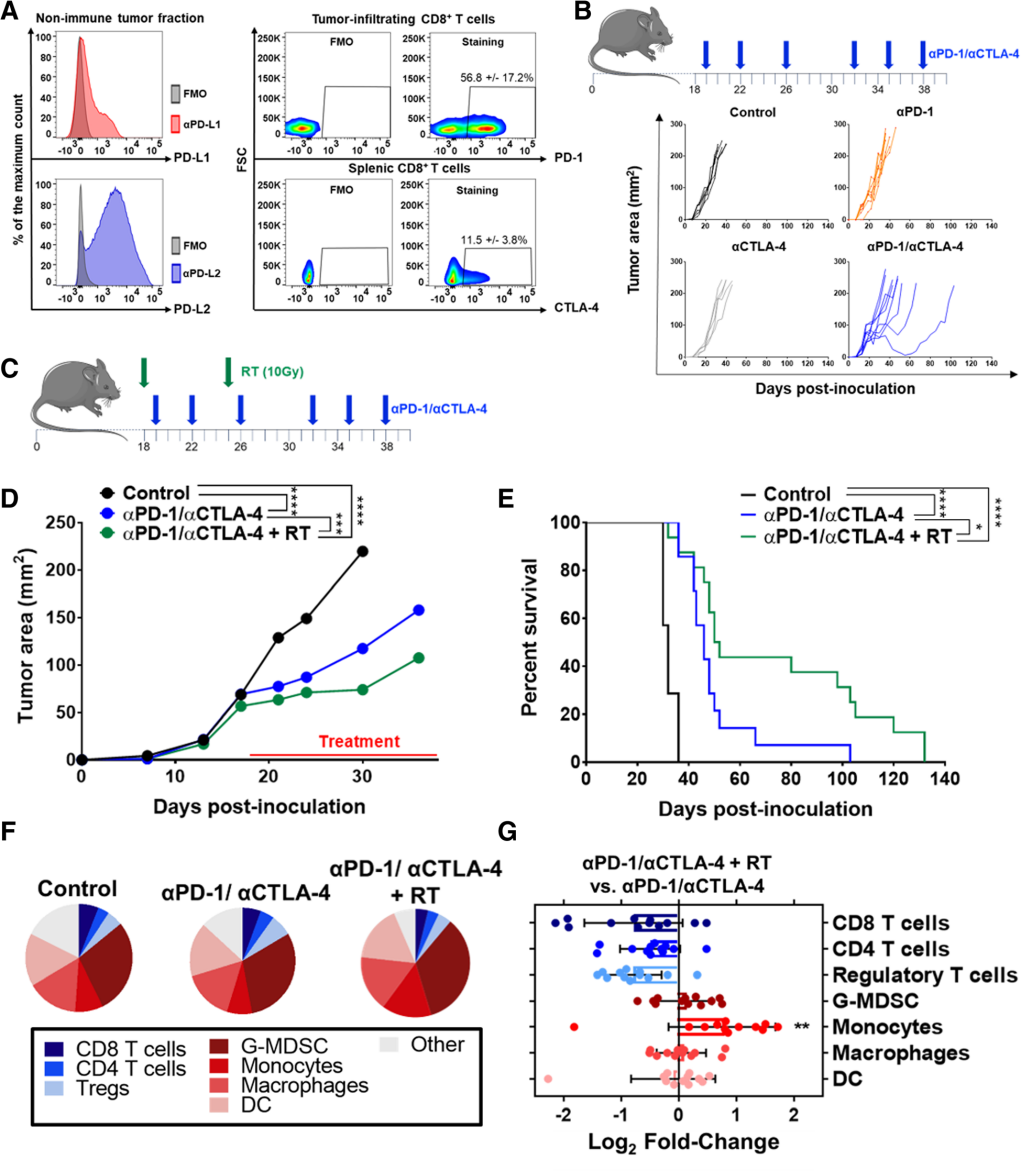
Immune checkpoint inhibition, with or without radiation, fails to clear established mEER tumors. **a** Flow cytometry immune profiling of untreated mEER tumors harvested at day 23 of tumor growth. Left shows a representative histogram for PD-L1 (top) and PD-L2 (bottom) within the non-immune tumor fraction (CD45 negative cells after gradient separation). Right shows cumulative flow cytometry scatterplots of PD-1 levels on tumor infiltrating CD8^+^ T cells (top) and CTLA-4 levels on splenic CD8^+^ T cells (bottom) (percentage show mean ^+^/− SD; *N* = 1 representative of 2; *n* = 5 aggregate samples per group). (**b** top) Subcutaneous established mEER tumors (day 17–18 post tumor cell injection) were treated with 6 total doses of αPD-1 (250 μg/dose) and/or αCTLA-4 (100 μg/dose). (**b** bottom) Individual tumor area for each ICI treated mouse subset (*N* = 1 representative of 2; *n* = 6–8/group). **c-e** Mice bearing established mEER tumors were treated with αPD-1 and αCTLA-4 alone or in combination with localized tumor irradiation (2 X 10 Gy with one dose given each week) according to the schedule in (**c**), and euthanized when tumors reached 225 mm^2^. **d** Average tumor area until time of first mouse euthanization (Tukey’s multiple comparison test; *N* = 1 representative of 2; *n* = 6–9/group). **e** Kaplan Meier curves comparing survival of mice treated with immune checkpoint inhibitors with and without tumor-directed irradiation (Log-rank test; *N* = 2; *n* = 12–18/group). **f** Pie-chart showing tumor-infiltrating lymphoid and myeloid subsets as a fraction of total CD45^+^ cells on day 23 of treatment (*N* = 2; *n* = 10–16/group). **g** Log2 fold-change of key immune subsets comparing αPD-1/αCTLA-4+ RT vs. αPD-1/αCTLA-4 at day 23 of treatment (Tukey’s multiple comparison test; *N* = 2; *n* = 10–12/group). *p < 0.05; **p < 0.01; ***p < 0.001; ****p < 0.0001

Many clinical studies have used intratumoral T cell expression of PD-1 and its cognate ligands, PD-L1 and PD-L2, as a correlate of treatment response [40–42] (clinical trials NCT03637491 and NCT03598270). As a result, we first characterized the nascent ICI response potential in the mEER tumor model by assessing expression of PD-1 axis molecules. In untreated mEER tumors within the non-immune (CD45 negative) fraction, flow cytometry demonstrated expression of both PD-L1 and PD-L2 (Fig. 1a). Further immune characterization revealed that over 50% of tumor infiltrating CD8^+^ T cells expressed PD-1 and over 10% of splenic CD8^+^ T cells expressed CTLA-4 (Fig. 1a). Interestingly, we did not observe detectable extracellular levels of CTLA-4 on intratumoral or tdLN-dwelling CD8^+^ T cells (Additional file 10: Figure S10A-B), potentially suggesting a lack of ongoing T cell priming and activation [43]. These data suggested that established mEER tumors may benefit from PD-1 and/or CTLA-4 inhibition using systemically delivered blocking antibodies (αPD-1 and αCTLA-4). To test this, mEER tumors were established for 17–18 days to a mean tumor area of 60 to 65 mm^2^ and provided αPD-1 (250 μg per dose) and/or αCTLA-4 (100 μg per dose) for a total of 6 doses (see Fig. 1b for treatment schematic). Surprisingly, αPD-1 and/or αCTLA-4 showed only minor tumor growth and survival improvements and even in combination remained incapable of promoting tumor rejection (Fig. 1b and Additional file 1: Figure S1A). Furthermore, TIME profiling using flow cytometry showed no significant differences in percentages of the predominant lymphoid and myeloid immune subsets (Additional file 1: Figure S1B; for flow gating strategy see Additional file 11: Figure S11 and Additional file 12: Figure S12). Additional assessment of the tdLN showed similar lymphocyte percentages for all ICI treated groups as well, with only minor increases in CD8^+^ T cell percentages for αCTLA-4 monotherapy treated mice (Additional file 1: Figure S1C). Collectively, these data suggest that αPD-1 and αCTLA-4, alone or in combination, promote only minor treatment benefit in established mEER tumors, likely due to their inability to overcome the highly immunosuppressive TIME.

### The combination of ICIs with radiation fails to reverse the “cold” tumor immune microenvironment

Radiation has been previously shown to stimulate a variety of immunologic effects that can improve ICI responses [13, 15–17]. Thus, we combined αPD-1 and αCTLA-4 (denoted as αPD-1/αCTLA-4) with tumor-directed radiation as a method to potentiate its therapeutic benefit. Mice bearing similarly established mEER tumors were treated with tumor-directed radiation delivered as 2 weekly 10 Gy fractions with concurrent αPD-1/αCTLA-4 treatment (see Fig. 1c for treatment schematic). Assessment of tumor growth and survival showed a significant treatment improvement in mice receiving αPD-1/αCTLA-4 and radiation compared to mice receiving ICIs alone (Fig. 1d and e; for individual tumor growth curves see Additional file 2: Figure S2A and B). Despite this improvement, the combinatory regimen remained incapable of promoting complete tumor regressions in this established tumor model. To better understand this limitation, we used flow cytometry at day 23 of treatment (5 days post-radiation) to characterize changes in the lymphoid and myeloid TIME. At this timepoint tumor sizes are similar between all treatment groups, thereby minimizing immunologic effects influenced by tumor size and allowing better comparison of treatment-related effects. As previously discussed, the TIME of αPD-1/αCTLA-4 treated tumors is very similar to that of untreated control tumors (Fig. 1f). The addition of radiation to αPD-1/αCTLA-4, appears to promote both lymphodepleting and general inflammatory effects as indicated by the modest decrease in various T cell subsets and a 1.8-fold increase in monocytic myeloid cells (Fig. 1g). Overall, these data suggest that even in the context of αPD-1/αCTLA-4 and tumor-directed radiation, the TIME remains relatively “cold”, with limited anti-tumoral immune cell infiltration and high levels of various immunosuppressive cell subsets such as granulocytic myeloid-derived suppressor cells (G-MDSC) and Tregs.

### CTX/L-NIL immunomodulation renders tumors responsive to the combination of αPD-1/αCTLA-4 and radiation (CPR)

**Fig. 2:**
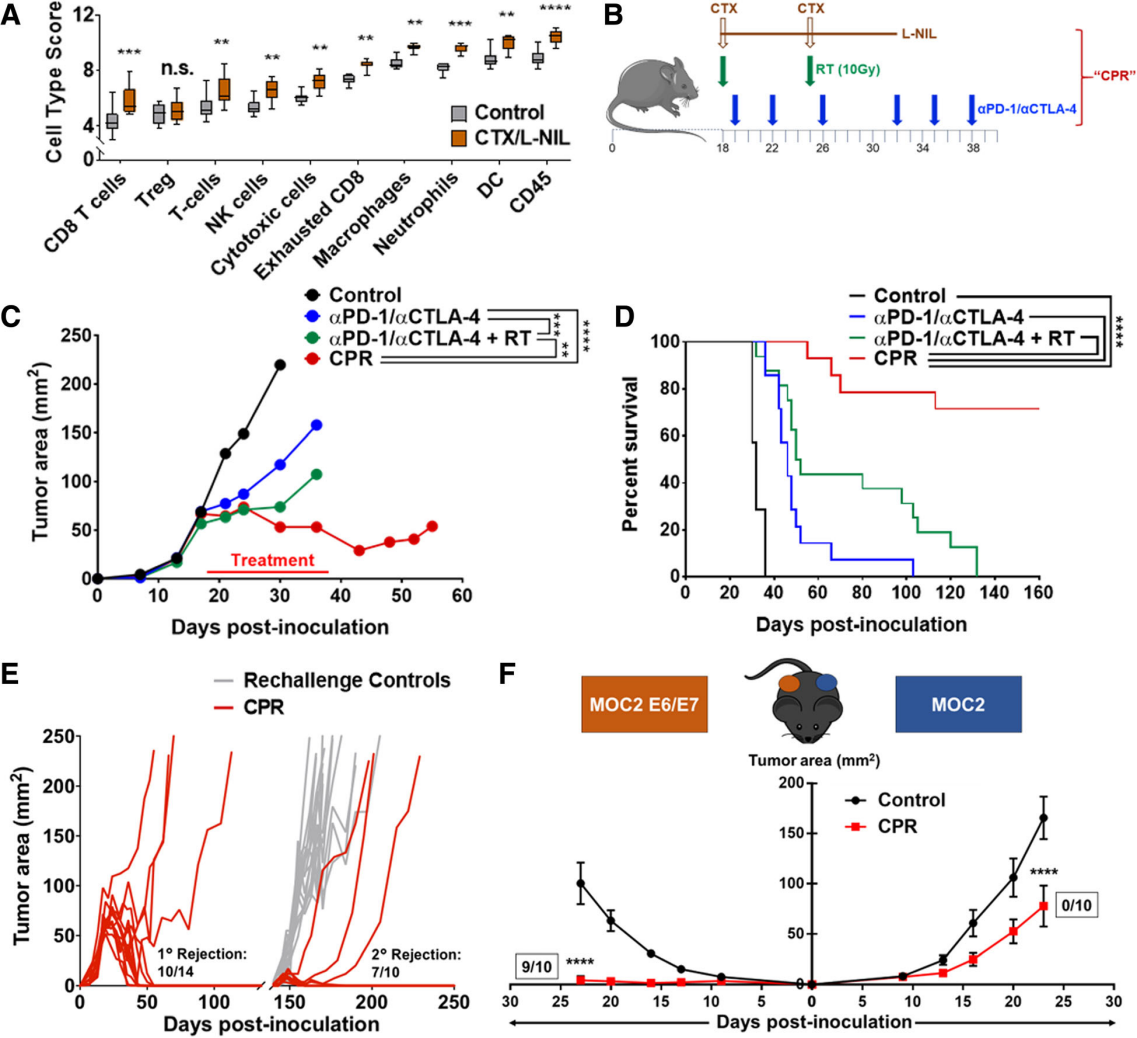
TIME modulation renders tumors responsive to αPD-1/αCTLA-4 with radiation and promotes immunologic memory. **a** Established mEER tumors were treated with CTX/L-NIL (2 doses of CTX at 2 mg/mouse delivered weekly and L-NIL 2 mg/mL continuously delivered in the drinking water). Tumor were harvested at day 23 of treatment and immune cell type enrichment scores from Nanostring whole tumor immune-related RNA expression was compared to untreated control tumors (Two-way ANOVA with Sidak correction; *N* = 1; *n* = 9/group). **c** and **d** Established mEER tumors were treated with CTX/L-NIL immunomodulation combined with αPD-1/αCTLA-4 and tumor directed radiation (collectively called the “CPR” regimen) according to schedule in (**b**), mice were euthanized when tumors reached 225 mm^2^. **c** Average tumor area until time of first mouse euthanization (Tukey’s multiple comparison test; *N* = 1 representative of 2; *n* = 6–8/group). **d** Kaplan Meier survival curves comparing different treatment combinations (Log-rank test; *N* = 2; *n* = 12–16/group). **e** CPR treated mice which rejected primary mEER tumor challenge were rechallanged approximately 100 days after primary rejection using 5-fold the original mEER tumor inoculum on the opposing flank. Data shows individual mouse tumor area compared to age-matched naïve control mice in gray (*N* = 2; *n* = 10/group). **f** Similar to 2E, CPR mice which rejected primary mEER tumor challenge were rechallanged simultaneously with MOC2 tumor cells and MOC2 tumor cells expressing HPV E6 and E7 on the opposing flank. Data shows average tumor area for MOC2 tumors (right) and MOC2 E6/E7 tumors (left) statistically compared to age-matched naïve control mice at time of first mouse euthanization (Tukey’s multiple comparison test; *N* = 2; *n* = 10/group). Fractions next to growth curves indicate the number of mice which fully rejected rechallange. **p < 0.01; ***p < 0.001; ****p < 0.0001, n.s. indicates not significant

We have previously shown that the combination of CTX (2 mg per mouse delivered weekly) and a selective small-molecular iNOS inhibitor, L-NIL (2 mg/mL continuously delivered in the drinking water for 2 weeks) favorably modulate the TIME [31, 32]. Immune gene expression profiling of tumors treated for 1 week with CTX/L-NIL reveals significant improvements in immune cell scores associated with anti-tumoral immune response, such as CD8^+^ T cells, dendritic cells (DCs), and cytotoxic cells (Fig. 2a); however, CTX/L-NIL treatment alone remains incapable of promoting complete remissions in established mEER tumors (Additional file 2: Figure S2B). One potential explanation is the significant increase in the gene expression signature for CD8^+^ T cell exhaustion (Fig. 2a) and the greater than 2-fold upregulation in PD-L1 and PD-L2 gene expression induced by CTX/L-NIL treatment compared to untreated controls (Additional file 2: Figure S2C). These data suggest that CTX/L-NIL immunomodulation could both benefit and be benefited by combination with αPD-1/αCTLA-4 and radiation. Thus, we developed a combinatory regimen delivering **C**TX/L-NIL immunomodulation, α**P**D-1/αCTLA-4 checkpoint inhibition, and **R**adiation (collectively called the “CPR” regimen; see Fig. 2b for treatment schematic). Upon treating similarly established mEER tumors, the CPR regimen significantly reduced tumor sizes over the course of treatment compared to αPD-1/αCTLA-4 with and without radiation (Fig. 2c). Long-term survival assessment further revealed that the CPR regimen promoted complete and stable tumor clearances in over 70% of treated mice, a significant improvement over all other groups (Fig. 2d). Assessment of gross toxicity through mouse weight reveals minor weight loss over the course of treatment (less than 10% of total body weight) with rapid recovery to control levels after treatment completion (Additional file 3: Figure S3A). Rejections remain stable for at least 100 days post-clearance and mice appeared healthy, with development of white fur patches near where the tumor was originally established, a typical observation in immune-related tumor clearances (Additional file 3: Figure S3B) [44]. To further assess the therapeutic potential of the CPR regimen we tested it in a secondary tumor model of B16 melanoma due to its well-reported resistance to ICI therapies and radiation, especially once established [13, 44, 45]. Using B16 we were further able to validate the treatment potential of this regimen, as the CPR regimen doubled the median survival time compared to αPD-1/αCTLA-4 and radiation (Additional file 4: Figure S4). These data suggest that the combination of CTX/L-NIL immunomodulation can safely and dramatically improve the treatment benefits of ICIs and radiation in solid tumors.

### CPR combination therapy promotes tumor antigen specific immunologic memory

Development of tumor-specific immunologic memory capable of long-term immune surveillance is a major theoretical benefit of cancer immunotherapies, and numerous reports suggest that ICIs can enhance this effect [46, 47]. Thus, we investigated whether the CPR regimen promoted development of tumor-specific memory. First, we assessed this using a tumor rechallenge approach, in which CPR treated mice were re-injected approximately 100 days after initial tumor clearance with 5-fold the original tumor inoculum in the opposing flank. We observed that 70% of mice that rejected the initial tumor challenge were capable of fully clearing the secondary tumor rechallenge, suggesting the development of tumor-specific immunologic memory (Fig. 2e). To further assess the antigen specificity of the immune memory response we utilized a dual flank rechallenge model using a HPV-negative HNSCC tumor model, MOC2, made with or without exogenous expression of E6 and E7 HPV antigens [35–37]. In CPR treated mice 100 days post-clearance, we re-challenged with parental MOC2 tumors on the initial tumor-bearing flank and MOC2 tumors transfected with E6 and E7 HPV viral oncoproteins (MOC2-E6/E7) on the opposing flank. We observed 90% complete clearance of the MOC2-E6/E7 tumors and minor, though significant, delays in the growth of MOC2 tumors lacking HPV antigen compared to age-matched control mice (Fig. 2f). These data suggest that the CPR regimen stimulated the development of potent immunologic memory to the original mEER tumor, including strong reactivity to E6 and E7 HPV viral antigens.

### CPR combination therapy promotes favorable changes in TIME and lymph node myeloid populations

**Fig. 3:**
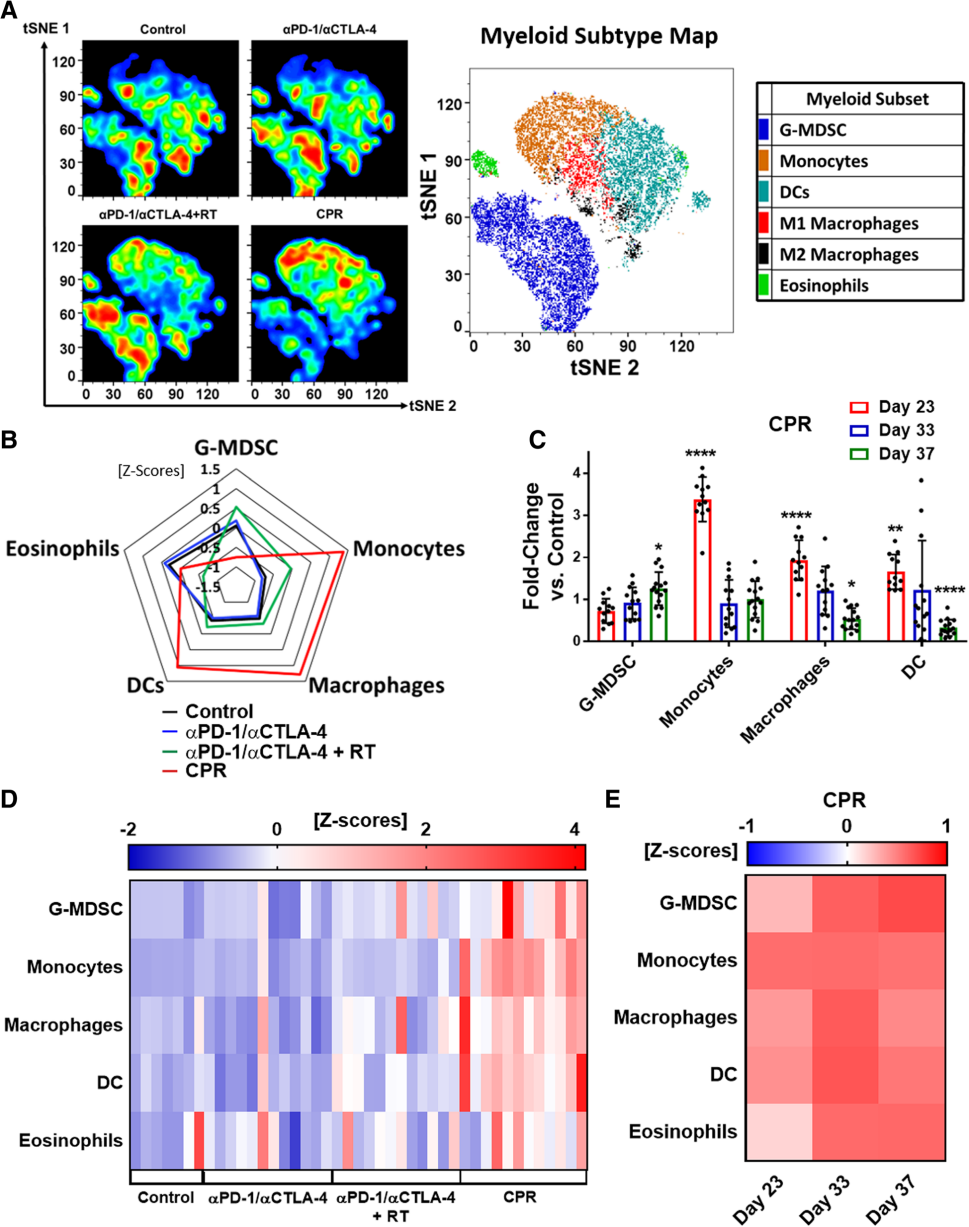
CPR favorably remodels the tumor and lymph node myeloid microenvironment. Mice bearing similarly established mEER tumors were treated and harvested after the first week of treatment (day 23) for assessment of myeloid cellular changes using flow cytometry in both the tumor (**a-c**) and the tdLN (**d** and **e**; see Additional file 11: Figure S11 for myeloid gating strategy). **a** Myeloid-focused tSNE (among intratumoral CD11b^+^ and/or CD11c^+^ cells) showing cumulative plots for each treatment group with corresponding myeloid subtype color map (right; *N* = 1 representative of 2; *n* = 5–6 per group). **b** Radar plot showing z-scores of myeloid sub-type percentages (among CD45^+^ cells) between treatment groups (*N* = 2; *n* = 10–12 per group). **c** CPR treated mice were assessed by flow at early (day 23), intermediate (day 33), and late (day 37) treatment timepoints and compared to tumor-size matched control mice for each of the myeloid subsets. Data shows fold-changes of intratumoral myeloid subtype percentages between CPR and control mice (Tukey’s multiple comparison test; *N* = 2; *n* = 11–13 per group, each dot represents an individual mouse). **d** Heatmap showing individual mouse z-scores for myeloid subtype percentage changes by treatment in the tdLN at day 23 of treatment (*N* = 2; *n* = 8–12 per group). **e** Heatmap showing average z-scores of myeloid subtypes for CPR treated mice compared to tumor-sized matched control mice (*N* = 2; *n* = 11–13 per group). *p < 0.05; **p < 0.01; ****p < 0.0001

To better characterize beneficial effects induced by the CPR regimen we assessed immunologic changes both within the tumor and tdLN at various timepoints of treatment; early (day 23), intermediate (day 33), and late (day 37) (see Fig. 2b for treatment schematic). Understanding the dynamics of the CPR regimen was crucial, especially since radiation has been shown to promote temporally restricted immune cell infiltration, typically between 5 and 10 days following treatment [48, 49]. Previously we reported that CTX/L-NIL treatment of established mEER tumors promoted a favorable shift within the myeloid TIME at early treatment timepoints [32]. Thus, we first wanted to investigate myeloid changes induced by the CPR regimen. Using t-distributed stochastic neighbor embedding (t-SNE) visualization of flow cytometry data, we observed broad alterations of tumor infiltrating myeloid cells at the early day 23 timepoint (visualized among CD11b^+^/CD11c^+^ myeloid cells; Fig. 3a). Qualitatively, the CPR regimen promotes intratumoral shifts away from immunosuppressive myeloid cell types, such as G-MDSC, to subsets associated with anti-tumor immune responses such as inflammatory monocytic cells, DCs, and macrophages (Fig. 3a). Quantification of this effect at day 23 of treatment shows significant increases in monocytes (3.3-fold), macrophages (1.9-fold), and DCs (1.6-fold) as well as a slight reduction in G-MDSC (1.3-fold reduction) in CPR treated tumors compared to tumor size-matched controls (Fig. 3b). Additionally, since macrophages can be polarized towards both antitumor (M1) and immunosuppressive (M2) phenotypes, we further classified the increase in total macrophages to be a predominantly M1-like phenotype based on high expression of MHCII and iNOS (Additional file 5: Figure S5). This early myeloid shift was unique to the CPR regimen and was not present following αPD-1/αCTLA-4 treatment alone or with radiation, suggesting that it is driven largely by the addition of CTX/L-NIL (Additional file 6: Figure S6D). Further analysis of CPR treated tumors at the intermediate (day 33) and late (day 37) treatment timepoints reveals a significant reduction in both macrophages (2-fold reduction) and DCs (3.5-fold reduction) by day 37 of treatment (Fig. 3c and Additional file 7: Figure S7D). We additionally note that the tdLN in CPR treated mice are similarly elevated in monocytes, macrophages, and DC at the early day 23 time point compared to all other groups (Fig. 3d and Additional file 8: Figure S8D). Unlike the tumor, the tdLN maintains elevated levels of each of these anti-tumor myeloid subsets over the full course of treatment compared to tumor size-matched controls (Fig. 3e and Additional file 9: Figure S9D). This suggests that the CPR treatment may be promoting migration and proliferation of myeloid cells into the draining lymph node where they stimulate further immune activation. These data demonstrate the favorable myeloid shift in the TIME and draining lymph node induced by the CPR regimen, which likely contribute to the improved treatment response induced by this combination.

### CPR combination therapy improves CD8^+^ T cell infiltration and activation

**Fig. 4:**
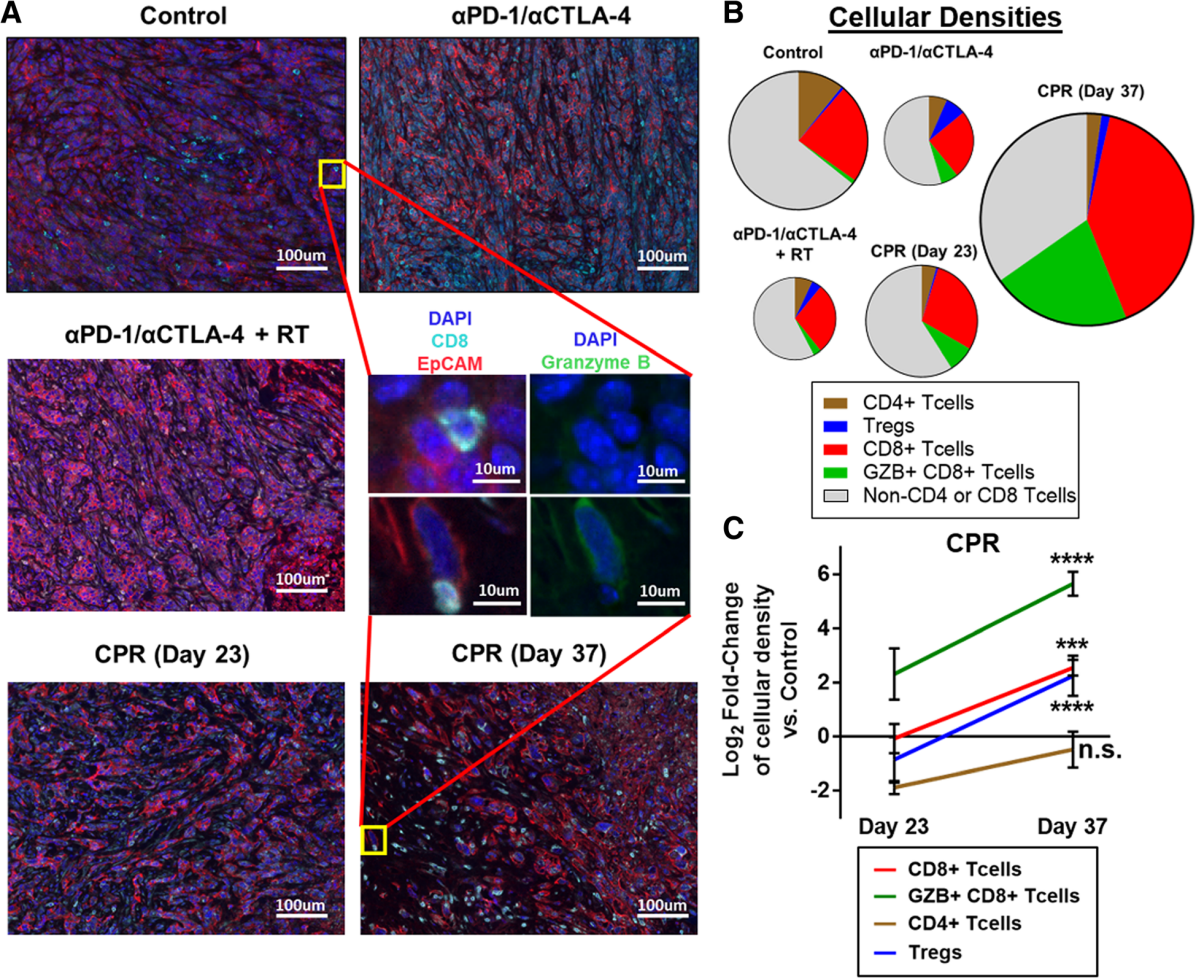
CPR treatment enhances intratumoral CD8^+^ T cell infiltration and activation**.** Established mEER tumors were treated with components of the CPR regimen and harvested on day 23 of treatment, or day 23 and day 37 for the full CPR regimen, and tumor lymphocyte infiltrates were analyzed using quantitative multiplex immunofluorescence. **a** Representative multiplex images of mEER tumors showing DAPI (nuclei, dark blue), EpCAM (tumor, red), and CD8 (CD8^+^ T cells, cyan). Zoomed middle insert shows a representative T cell from a control and day 37 CPR treated tumors with DAPI/EpCAM/CD8 stain on left and DAPI/Granzyme B (activated T cell marker, green) on right. **b** Pie-chart showing T cell subset densities as a fraction of the whole T cell tumor infiltrate by treatment group. Pie area corresponds to the total T cell density per treatment group. **c** Log2 fold-change of lymphocyte subset densities (counts per total nucleated cells) in CPR tumors vs control tumors statistically comparing day 23 and day 37 of CPR treatment (Tukey’s multiple comparison). For all samples *N* = 1 and cellular densities were averaged across 5 images per tumor with *n* = 3 per group. ***p < 0.001; ****p < 0.0001

Due to the improved myeloid composition in the TIME, we next assessed whether this treatment promoted changes in tumor lymphocyte infiltration and activation using quantitative immunofluorescent imaging. Qualitatively we observed that tumors treated with ICIs with or without radiation at day 23 of treatment had minimal CD8^+^ T cell infiltration and were largely characterized by densely packed regions of tumor cells (as denoted by EpCAM expression; Fig. 4a). Interestingly, CPR treated tumors at day 23 have a distinct appearance, with approximately 75% the density (per nucleated cell) of tumor cells compared to ICI treatment alone and a high infiltration of non-T cell (CD3^−^) immune cells, consistent with our previous data suggesting that CPR treated tumors are largely myeloid infiltrated at early treatment timepoints. By day 37 of CPR treatment, a striking increase in the number of CD8^+^ T cells, and granzyme B expression is observed (Fig. 4a). Quantification of the various T cell subsets reveals that all treatment groups at day 23 appear depleted in total T cells (counts per total nucleated cell) compared to control tumors (Fig. 4b). However, further quantification at day 23 reveals that while the CPR regimen promotes levels of CD8^+^ T cell densities similar to control tumors, it stimulates a 4-fold increase in activated granzyme B-expressing CD8^+^ T cells (Fig. 4c). At day 37 of CPR treatment we observe a substantial expansion of these subsets, with a 4-fold increase in CD8^+^ T cell density and a greater than 30-fold increase in Granzyme B expressing CD8^+^ T cell density (Fig. 4c; see Additional file 13: Figure S13 for raw cellular densities). Overall these data suggest that the CPR regimen stimulates CD8^+^ T cell infiltration and activation, especially at later treatment timepoints, a likely result of the beneficial myeloid TIME and tdLN alterations.

### CPR combination therapy stimulates proliferation, tumor infiltration, and activation of CD8^+^ T cells

**Fig. 5:**
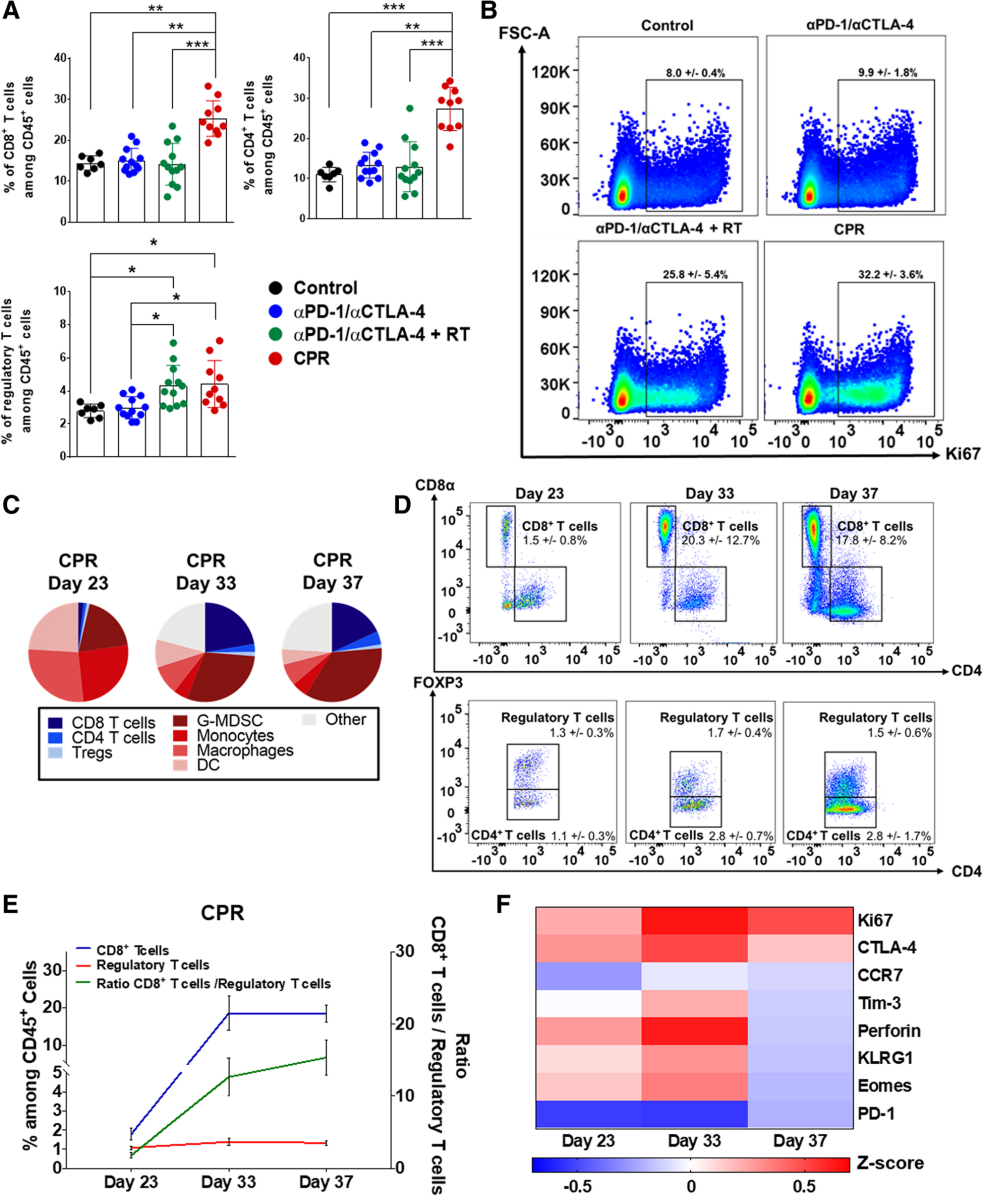
CPR treatment stimulates T-cell proliferation, activation, and improves lymphoid effector-to-suppressor ratios. Mice bearing established mEER tumors were harvested after 1 week of treatment (day 23) for assessment of lymphoid cellular changes using flow cytometry both in tdLN (**a** and **b**) and tumor (**c-f**; see Additional file 12: Figure S12 for lymphoid gating strategies). **a** Percentage of lymphoid subsets within the tdLN (among CD45^+^ cells; Dunn’s multiple comparison test; *N* = 2; *n* = 7–12 per group). **b** Aggregate flow cytometry scatterplots showing Ki67 expression among CD8^+^ T cells within the tdLN (percentages show mean ^+^/− SD; *N* = 1 representative of 2; *n* = 6 aggregate samples per group). **c** Pie-chart showing average tumor-infiltrating lymphoid and myeloid subsets as a fraction of total CD45^+^ cells for CPR treated tumors at days 23, 33, and 37 (*N* = 2; *n* = 10–16/group). **d** Aggregate flow cytometry scatter plots of CPR treated tumors showing CD8^+^ T cells (top panels), CD4^+^ T cells and regulatory T cells (bottom panels) at each day of treatment progression (percentages show mean ^+^/− SD; *N* = 1, representative of 2; *n* = 6 aggregate samples per day). **e** Summary of CPR intratumoral CD8^+^ and regulatory T cell percentages (among CD45^+^ cells; left y-axis) and the ratio of CD8^+^ T cell / regulatory T cells (right y-axis) at days 23, 33, and 37 of treatment (*N* = 2; *n* = 10–16/group). **f** Intratumoral CD8^+^ T cell phenotypic marker expression at days 23, 33, and 37 of CPR treatment progression. Data is represented as a z-score of the phenotypic marker’s median fluorescence intensity (MFI) compared to size-matched control tumors (*N* = 2; *n* = 11–13 per group). *p < 0.05; **p < 0.01; ***p < 0.001

To further characterize the lymphoid effects induced by the CPR treatment we used flow cytometry to profile both the tumor and tdLN. Assessment of the tdLN at the early day 23 of treatment showed a unique T cell proliferation effect in CPR treated mice. This includes significant increases in the percentages of CD8^+^ T cells (1.7-fold), CD4^+^ T cells (2.2-fold), and a minor increase in Tregs (1.4-fold) compared to both control and αPD-1/αCTLA-4 treated mice (Fig. 5a and Additional file 8: Figure S8A and C). Further characterization of lymphocyte proliferation (as indicated by Ki67 expression) revealed a 3-fold increase in Ki67 expressing CD8^+^ T cells within the tdLN of CPR treated mice compared to both control and αPD-1/αCTLA-4 treated mice (Fig. 5b). This effect appears at least partially due to the addition of radiation to αPD-1/αCTLA-4, as it more than doubled Ki67 expressing CD8^+^ T cells compared to control groups as well (Fig. 5b). This enhancement in tdLN lymphoproliferation was noted at each day of CPR treatment, as we observed increased lymphocyte percentages and Ki67 expression at days 23, 33, and 37 of treatment compared to tumor size-matched control mice (Additional file 8: Figure S8A, Additional file 9: Figure S9A and Additional file 10: Figure S10).

Due to the favorable lymphoproliferation effects within the lymph node, we next performed lymphocyte subset analysis within tumors over the same treatment time-course. Early (day 23) time-point analysis of CPR treated tumors showed a largely myeloid-dominated tumor, with small and approximately equal fractions of CD8^+^, CD4^+^, and Tregs (Fig. 5c). However, at the intermediate (day 33) and late (day 37) timepoints, we observed more than a 13-fold increase in the percentage of CD8^+^ T cells and a greater than 2-fold increase in CD4^+^ T cells infiltrating CPR treated tumors compared to tumor size-matched control mice (Fig. 5d). We additionally observed consistently low levels of tumor infiltrating immunosuppressive Tregs over the full course of treatment, which contributed to the 15-fold improvement in the CD8^+^ T cell to Treg ratio (Fig. 5e and Additional file 8: Figure S8A and C). Based on a consensus nomenclature for CD8^+^ T cell phenotypes [50], phenotyping of tumor infiltrating CD8^+^ T cells at each day of CPR treatment revealed a strongly proliferating (i.e. Ki67^+^) CD8^+^ T cell subset expressing numerous molecules associated with both effector (i.e. Perforin, killer cell lectin-like receptor-KLRG1) and memory (i.e. Eomes, low PD-1) T cell status (Fig. 5f) [51]. Towards the end (day 37) of treatment CD8^+^ T cells appear to have entered a late stage of tumor killing due to the loss of numerous effector markers including Eomes and perforin (Fig. 5f; see Additional file 10: Figure S10 for CD8^+^ T cell phenotypes for all groups and tissues) [52], and further supported by the fact that tumors rapidly regress and clear between days 37 to 50 (see Fig. 2c and e). In addition, we observed elevations in E7 specific CD8^+^ T cells by E7 tetramer staining both in the tumor and tdLN of CPR treated mice at days 33 and 37 of treatment (Additional file 7: Figure S7B and Additional file 9: Figure S9B) but only observed minor levels at day 23 for any groups (Additional file 6: Figure S6B and Additional file 8: Figure S8B; for representative tetramer staining see Additional file 6: Figure S6E, Additional file 7: Figure S7E, Additional file 8: Figure S8E, and Additional file 9: Figure S9E). Overall, these observations suggest the CPR regimen is capable of activating the lymphoid TIME at least partially by driving strong T cell proliferation in both the tumor and tdLN; limiting intratumoral infiltration and development of Tregs; and enhancing the activation status and specificity of tumor-infiltrating CD8^+^ T cells.

### CD8^+^ T cells are necessary for tumor clearance after CPR combination treatment

**Fig. 6:**
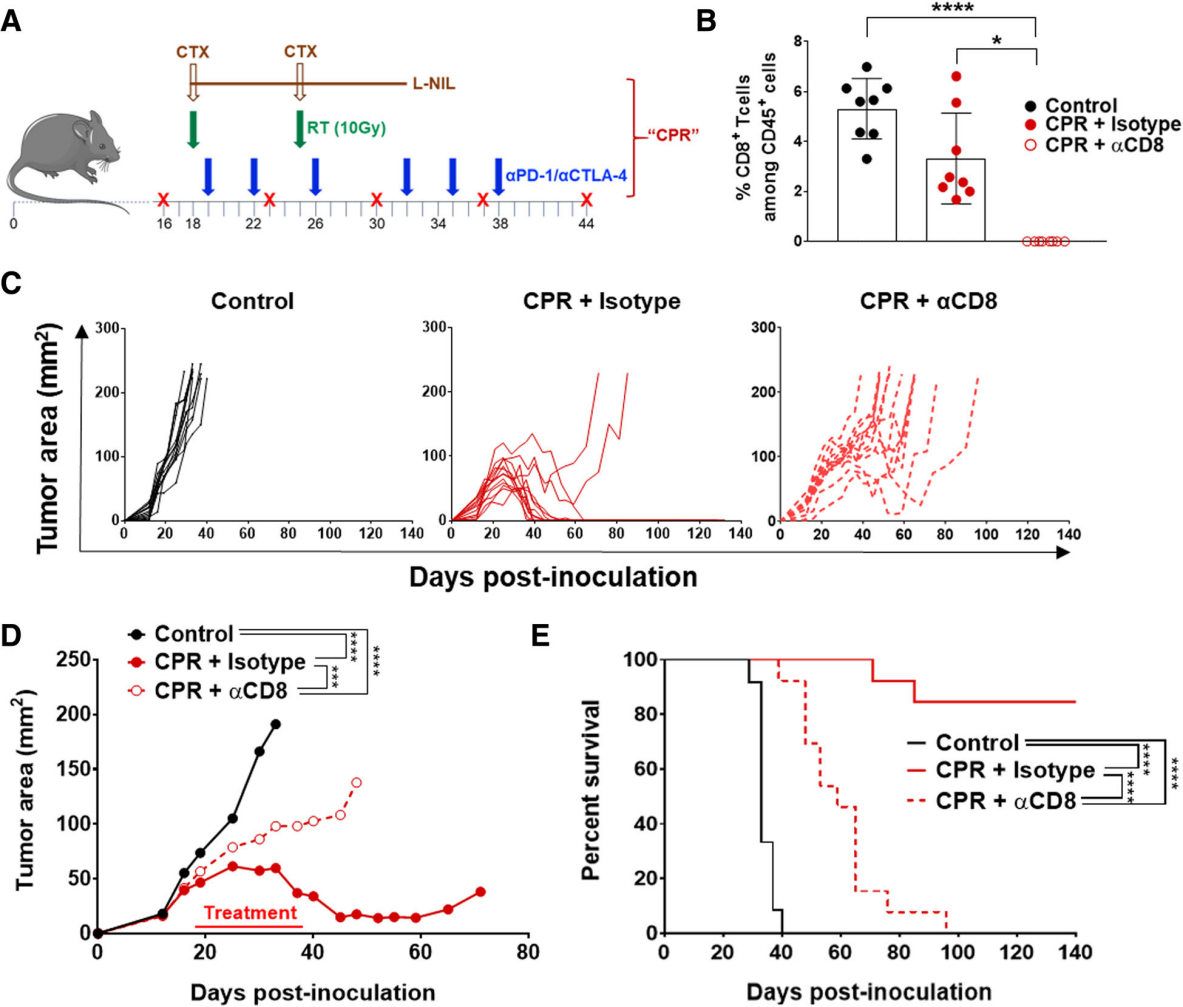
CD8^+^ T cells are necessary for tumor clearance after CPR. Established mEER tumors were treated with CPR and anti-CD8α depleting antibody, or isotype control antibody, according to the schedule in (**a**); mice were euthanized when tumors reached 225 mm^2^. **b** CD8^+^ T cell percentages (among CD45^+^ cells) in the blood at day 33 of treatment as assessed by flow cytometry (Dunn’s multiple comparison test; *N* = 1; *n* = 8 per group, each as an individual dot). **c** Individual tumor area by treatment group, with each mouse represented as a single line. **d** Average tumor area with statistical comparison at time of first control mouse euthanization (Tukey’s multiple comparison test; *N* = 1 representative of 2; *n* = 8 per group). **e** Kaplan Meier survival curves and statistical comparison between treatment groups (Log-rank test; *N* = 2; *n* = 12–13). *p < 0.05; ***p < 0.001; ****p < 0.0001

Both chemotherapy and tumor-directed radiation, components of the CPR regimen, are well-known to have immune-independent treatment effects [53, 54]. Thus, we wanted to validate the role of the immunologic response induced by the CPR regimen through cellular depletion studies. Due to the pronounced CD8^+^ T cell effects observed in the full CPR treatment regimen, we depleted CD8^+^ T cells using a CD8 targeting antibody delivered weekly throughout CPR treatment in similarly established mEER tumors (see Fig. 6a for depletion schedule). Effective CD8^+^ T cell depletion was validated in the blood of mice at the intermediate day 33 treatment timepoint. We observed a near-complete depletion of circulating CD8^+^ T cells to less than 0.1% the levels of both control and CPR treated mice administered isotype antibody (Fig. 6b). Assessment of tumor growth showed a significant increase in tumor sizes in CPR treated mice depleted of CD8^+^ T cells following treatment compared to non-depleted CPR mice (Fig. 6c and d). Additionally, CPR treated mice depleted of CD8^+^ T cells appeared unable to fully clear their tumor, and as a result have significantly decreased survival (Fig. 6e). Interestingly, CPR treatment in the absence of CD8^+^ T cells still promotes significant tumor growth delays and survival benefit compared to isotype treated control mice (Fig. 6c-e). This supports the notion that the chemoradiotherapy components of this regimen, and likely other immunologic cellular subsets, are also contributing to the treatment benefit of the CPR regimen. Collectively, these data suggest that although the CPR regimen may promote some non-immune related treatment effects, its ability to induce complete tumor clearance is entirely dependent on its immunologic effects, particularly the induction and activation of CD8^+^ T cells.

## Discussion

**Fig. 7:**
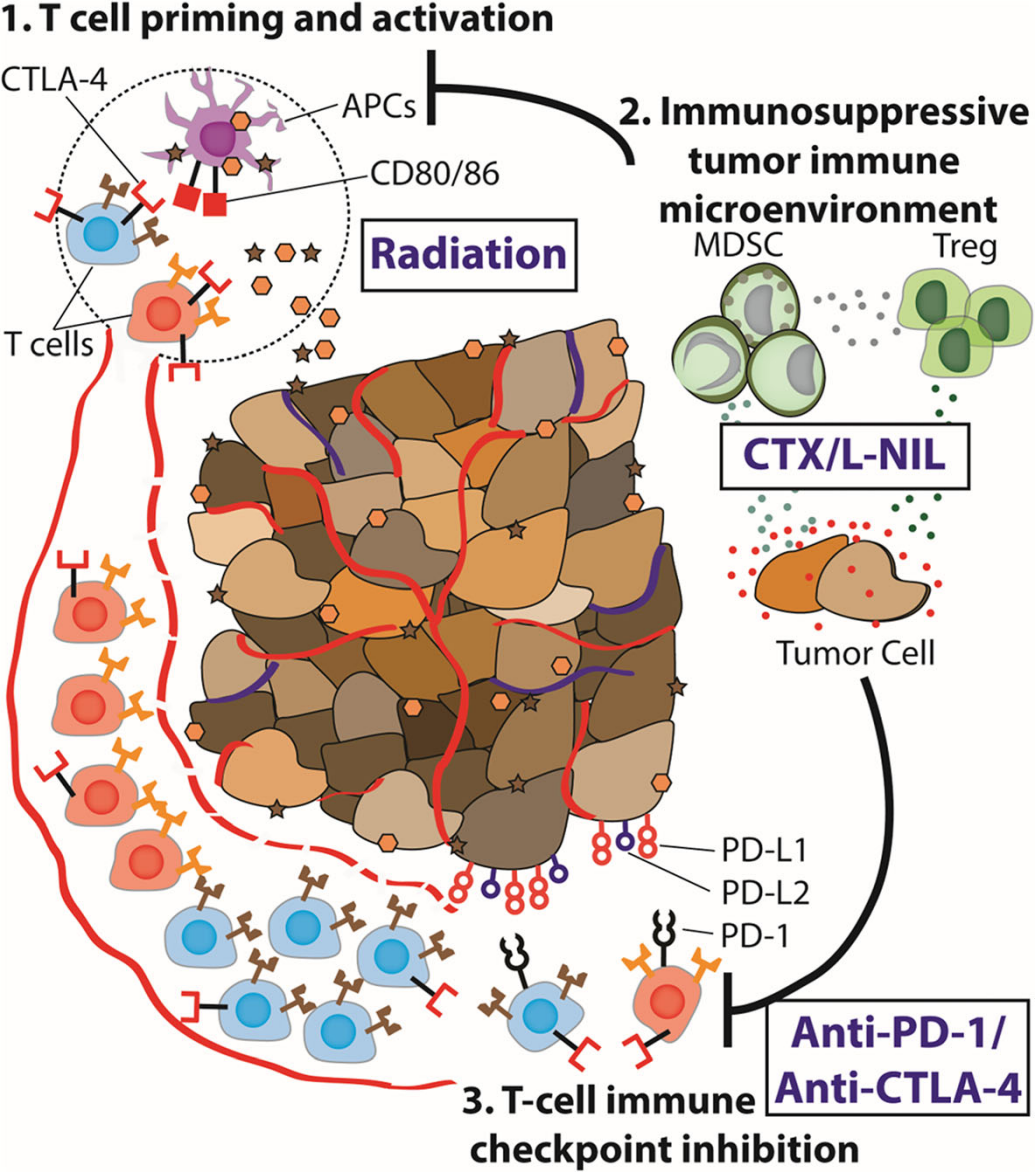
Immune microenvironment modulation unmasks therapeutic benefit of radiotherapy and checkpoint inhibition. Schematical abstract: Radiation provides potent tumor myeloid and APC infiltration and lymphoid stimulation in the tumor draining lymph node, however, the tumor immune microenvironment often remains immunosuppressed or immunologically “cold”. Targeting of the tumor immune microenvironment using CTX/L-NIL reverts the “cold” intratumoral microenvironment, providing an enhanced myeloid and lymphoid tumor and tdLN microenvironment. Thus, when CTX/L-NIL is combined with radiation and αPD-1/αCTLA-4 inhibition it allows potent immunologic rejection of established tumors and the development of tumor-antigen specific memory

In this study, we demonstrate the pivotal role of the TIME in limiting efficacy of ICIs and radiation, and further describe an effective immunomodulatory approach combining CTX and a selective small-molecule iNOS inhibitor, L-NIL, to revert its adverse effects. When CTX/L-NIL was combined with ICIs and radiation (the CPR regimen), it reversed the immunosuppressive TIME, leading to complete tumor clearance and the development of tumor-antigen specific memory in over 70% of mice bearing large, established tumors. While other studies have demonstrated the therapeutic benefit of modulating the TIME, immune characterization was often performed at a single timepoint and typically focused on specific immune cell types (i.e. T cells) [28, 55, 56]. Using flow cytometry and immunofluorescence imaging, we comprehensively profiled both myeloid and lymphoid immune microenvironment alterations induced by the CPR regimen at multiple treatment timepoints within the tumor and tdLN. These studies revealed broad and temporally-restricted alterations in the myeloid immune microenvironment, leading to significantly improved intratumoral lymphocyte infiltration at later timepoints, including a greater than 15-fold increase in the CD8^+^ T cell to Treg ratio. Overall, our results provide a clear example of effective TIME modulation, which could potentially be used to evaluate other exploratory immunomodulatory strategies (Fig. 7).

Our previous and current evidence of the immunomodulatory potential of CTX [31, 32] are consistent with a wide-body of literature describing its immune stimulating effects [57]. Examples include its ability to decrease Treg levels [58], increase cytotoxic T cell activity [59–61], and enhance APC activation [62]. As a result, CTX has been proposed as a potential combination with ICIs; however, few studies have directly tested this approach in preclinical models [14, 63, 64]. To further drive beneficial immunomodulation, we combined CTX with selective iNOS inhibition using L-NIL [32]. iNOS has been implicated in a variety of immunosuppressive and therapeutic resistance mechanisms in solid tumors [65, 66] and as a critical mediator in the recruitment and suppressive function of G-MDSC [30]. Results from this study further suggest that CTX and L-NIL in combination not only function as a potent immunomodulator to target immunosuppressive cells types commonly associated with solid tumors (i.e. Tregs and G-MDSC), but also stimulate the generation, infiltration, and activation of both myeloid and lymphoid anti-tumor immune subsets. Additionally, these results further validate the importance of combination therapies targeting both myeloid and lymphoid tumor compartments, as both of these compartments are now well known to play a role in ICI efficacy [67]. This has been further suggested by various reports which show enhanced ICI efficacy when combined with myeloid-focused therapies including combinations of GM-CSF activated DCs loaded with tumor-antigen [44, 68] stimulator of interferon genes (STING) vaccine strategies [69], and indolamine 2,3 dioxygenase inhibitors [29, 70].

Due to the expansive number ICI clinical trials in combination with tumor-directed radiation and/or chemotherapeutics, the translational implications of our findings are significant [71]. Our findings not only demonstrate potential readouts of tumor ICI reactivity but also provide recommendations for treatment schedule design. Nevertheless, further investigation is required prior to clinical implementation of the full CPR regimen. The first is a better understanding of the dosing and schedule for CTX, which has been shown to strongly influence its immunologic effects [72, 73]; however, despite numerous prospective and retrospective clinical reports, the optimal CTX dosing schedule for immunomodulatory benefit remains unclear [57]. Similar to CTX, the optimal radiation schedule remains similarly uncertain and requires further investigation. During these studies, we investigated alterative radiation schedules within the CPR regimen and observed that hypo-fractionated “stereotactic body radiotherapy (SBRT)-like” schedules (higher dose with fewer fractions) provide optimal treatment benefit in the mEER tumor model compared to more fractionated regimens; however, further assessment in B16 melanoma models generated opposing results (data not shown). This reflects the existing literature, in which conflicting reports claim enhanced immunomodulatory benefit using different radiation dosing strategies [74–76]. A final translational hurdle relates to the iNOS inhibitor used in this study, L-NIL. Although L-NIL has been assessed in clinical trials for inflammatory diseases [77], it requires additional study before utilization as a cancer therapeutic. Nevertheless, iNOS inhibition has been demonstrated using other clinically available drugs such as phosphodiesterase 5 inhibitors (i.e. sildenafil and tadalafil) and doxycycline [78–80], which may provide an alternative for faster translation to clinical trials.

Overall, our results provide a broad immunologic investigation of the factors in the TIME which limit response to ICIs and radiotherapy, and demonstrate that their reversal with the CPR regimen greatly enhances treatment efficacy. One potential limitation of our study is the absence of more thorough cellular characterization using additional markers of activation status and cellular sub-types. For example, B cells are known to be present at elevated levels in tdLN of tumor bearing mice, yet their role as pro- or anti-tumor remains unclear [81, 82]. Upon treatment, we did observe significant B cell depletion, which may have contributed to the treatment efficacy, however, we were unable to determine whether this plays a role in treatment responsiveness (data not shown). Additionally, there exist numerous sub-classes of the various cell types we detail in this study. For example, among DCs, plasmacytoid DCs (pDC) are potent inducers of Th1 immune responses [83, 84] and our prior gene-expression analysis suggested that pDCs may be upregulated following CTX/L-NIL immunomodulation [32]. Thus, future studies will be necessary to more thoroughly characterize the full immune landscape of this immunomodulatory treatment combination.

A final limitation of our study exists in the lack of analysis in primary patient samples, which remains challenging due to current capabilities of ex vivo model systems. Despite some recently published methods which can provide a more accurate representation of the tumor microenvironment [85], a major advantage compared to most ex vivo systems, these platform remain incapable of recapitulating the systemic consequences of radiation [76]. In addition, these systems fail to recapitulate the tumor-tdLN interaction which we found to be a critical feature of the CPR regimen. Therefore, future work will focus on alternative methods to assess the translatability of the CPR regimen in primary patients samples and data.

## Conclusions

In conclusion, multi-component remodeling of the TIME has the potential to significantly expand the fraction of patients responding to ICI and radiation therapies. We believe that the clinical relevance of these findings and the therapeutic interventions used could potentially be applied to diverse solid tumor malignancies where the immunosuppressive TIME impedes effective anti-tumor immunologic responses.

## Additional files

Additional file 1: **Figure S1.** Immune checkpoint inhibitors alone provide minimal treatment benefit or microenvironment improvement. (**A**-**C**) Subcutaneous established mEER tumors (day 17–18 post tumor cell injection) were treated with 6 total doses of αPD-1 (250 μg/dose) and/or αCTLA-4 (100 μg/dose) according to the schedule in (Fig. 1b), mice were euthanized when tumors reached 225 mm^2^. (**A**) Kaplan Meier survival curves with comparison between treatment groups (Log-rank test; *N* = 1 representative of 2; *n* = 5–12). (**B**) Flow cytometry immune profiling of treated tumors at 1 week of treatment (day 23) shown as z-scores for major lymphoid and myeloid subtypes between groups (*N* = 2; *n* = 9–12 per group). (**C**) Percentage of major lymphoid subsets (among CD45^+^ cells) in tdLN at day 23 of treatment for various treatment groups (Tukey’s multiple comparison test; *N* = 2; *n* = 9–12 per group). *p < 0.05; **p < 0.01; ***p < 0.001; ****p < 0.0001. (PDF 2648 kb)

Additional file 2: **Figure S2.** Individual tumor growth curves for singlet and dual treatments and CTX/L-NIL gene expression. Subcutaneous established mEER tumors (day 17–18 post tumor cell injection) were treated with individual or dual treatment combinations of PD-1/CTLA-4, CTX/L-NIL, and radiation (RT) according to the same schedule shown in Figs. 1c and 2b. (**A**) Individual mEER tumor growth curves for 2 experiments, one of which was used for in Fig. 1d (*N* = 2; *n* = 7–17 per group). (**B**) Individual tumor growth curves for singlet and dual treatment combinations of CPR regimen (*N* = 2–3; *n* = 12–19). (**C**) Differential gene expression of CTX/L-NIL treated tumors compared to control tumors compared after 1 week (day 23) of treatment with PD-L1 and PD-L2 noted in red dots (*N* = 1; *n* = 9 per group). Blue lines indicate gene 2-fold change point (vertical) and corrected *p*-value less than 0.0001 (horizontal). (PDF 3329 kb)

Additional file 3: **Figure S3.** CPR regimen induces minimal weight loss and no gross treatment related toxicities. (**A**) Normalized weight for treated mice over the course of treatment, normalized to mouse weight 1 week after tumor cell inoculation (*N* = 1 representative of 2; *n* = 5–9). (**B**) Image of mouse treated with full CPR regimen approximately 100 days after tumor clearance with white fur visible in location of tumor clearance. (PDF 1600 kb)

Additional file 4: **Figure S4.** CTX/L-NIL improves anti-tumor effect of αPD-1/αCTLA-4 and radiation in the B16 syngeneic melanoma tumor model. Subcutaneous established B16-F0 melanoma tumors (day 4 post tumor cell injection) were treated with αPD-1/αCTLA-4 and radiation alone, or combined with CTX/L-NIL immunomodulation (CPR regimen), mice were euthanized when tumors reached 225 mm^2^. (**A**) Average tumor area statistically compared at time of first control mouse euthanization (Tukey’s multiple comparison test; *N* = 1 representative of 2; *n* = 7–8 per group). (**B**) Kaplan Meier survival curves with comparison between treatment groups (Log-rank test; *N* = 2; *n* = 10–11 per group). *p < 0.05; ****p < 0.0001. (PDF 1425 kb)

Additional file 5: **Figure S5.** CPR increases intratumoral M1-like macrophages. Aggregate flow cytometry scatterplots showing MHCII and iNOS expression among tumor-dwelling macrophages at day 23 of treatment (percentages show mean ^+^/− SD; *N* = 1 representative of 2; *n* = 4 aggregate samples per group). (PDF 1299 kb)

Additional file 6: **Figure S6.** Tumor immune microenvironment data at day 23. Flow cytometry assessment of tumor was performed at day 23 for all treatment groups and major immune cell subset percentages (among CD45^+^ cells) are shown. (**A**) Percentage of CD4^+^ and CD8^+^ T cell subsets. (**B**) Percentage E7 tetramer^+^ CD8^+^ T cells. (**C**) Percentage of Tregs. (**D**) Percentage of major myeloid subsets. (For **A-D**, Tukey’s multiple comparison test; *N* = 2; 8–13 per group). (**E**) Aggregate flow cytometry scatter plots of CD8^+^ T cells showing E7 tetramer staining (*N* = 1, representative of 2; *n* = 4 aggregate samples per group). *p < 0.05; **p < 0.01; ***p < 0.001; ****p < 0.0001. (PDF 15352 kb)

Additional file 7: **Figure S7.** Tumor immune microenvironment data time course. Flow cytometry assessment of tumor was performed at day 23, day 33, and day 37 for the CPR treatment group and major immune cell subset percentages (among CD45^+^ cells) are shown. (**A**) Percentage of CD4^+^ and CD8^+^ T cell subsets. (**B**) Percentage E7 tetramer^+^ CD8^+^ T cells. (**C**) Percentage of Tregs. (**D**) Percentage of major myeloid subsets. (For **A-D**, Tukey’s multiple comparison test; *N* = 2; 8–13 per group). (**E**) Aggregate flow cytometry scatter plots of CD8^+^ T cells showing E7 tetramer staining (*N* = 1, representative of 2; *n* = 4 aggregate samples per group). **p < 0.01; ***p < 0.001; ****p < 0.0001. (PDF 14226 kb)

Additional file 8: **Figure S8.** tdLN immune microenvironment data at day 23. Flow cytometry assessment of tdLN was performed at day 23 for all treatment groups and major immune cell subset percentages (among CD45^+^ cells) are shown. (**A**) Percentage of CD4^+^ and CD8^+^ T cell subsets. (**B**) Percentage E7 tetramer^+^ CD8^+^ T cells. (**C**) Percentage of Tregs. (**D**) Percentage of major myeloid subsets. (For **A-D**, Tukey’s multiple comparison test; *N* = 2; 7–13 per group). (**E**) Aggregate flow cytometry scatter plots of CD8^+^ T cells showing E7 tetramer staining (*N* = 1, representative of 2; *n* = 4 aggregate samples per group). *p < 0.05; **p < 0.01; ***p < 0.001; ****p < 0.0001. (PDF 15328 kb)

Additional file 9: **Figure S9.** tdLN immune microenvironment data time course. Flow cytometry assessment of tdLN was performed at day 23, day 33, and day 37 for the CPR treatment group and major immune cell subset percentages (among CD45^+^ cells) are shown. (**A**) Percentage of CD4^+^ and CD8^+^ T cell subsets. (**B**) Percentage E7 tetramer^+^ CD8^+^ T cells. (**C**) Percentage of Tregs. (**D**) Percentage of major myeloid subsets. (For **A-D**, Tukey’s multiple comparison test; *N* = 2; 7–13 per group). (**E**) Aggregate flow cytometry scatter plots of CD8^+^ T cells showing E7 tetramer staining (*N* = 1, representative of 2; *n* = 4 aggregate samples per group). *p < 0.05; **p < 0.01; ***p < 0.001; ****p < 0.0001. (PDF 14769 kb)

Additional file 10: **Figure S10.** CD8^+^ T cell phenotype microenvironment heatmaps. Flow cytometry assessment of CD8^+^ T cell phenotype changes at day 23 for all treatment groups, and further assessed at day 33 and 37 for CPR treatment and compared to tumor size-matched control mice. Data is represented as a z-score of the phenotypic marker’s median fluorescence intensity (MFI) among CD8^+^ T cells represented as z-scores. (**A**) Tumor and (**B**) tdLN CD8^+^ T cell phenotypic marker expression between all treatment groups at day 23 of treatment (*N* = 2, *n* = 7–13 per group). (**C**) tdLN CD8^+^ T cell phenotypic marker expression at days 23, 33, and 37 of CPR treatment progression compared to size-matched control tumors (*N* = 2; *n* = 7–13 per group). (PDF 3118 kb)

Additional file 11: **Figure S11.** Myeloid flow cytometry gating strategy. Flow cytometry strategy used for gating myeloid subsets, with labeled cell type above the respective gate. (PDF 2002 kb)

Additional file 12: **Figure S12.** Lymphoid flow cytometry gating strategies. Flow cytometry strategy used for gating lymphoid subsets, with labeled cell type above the respective gate. (**A**) T cell phenotype panel which included various markers to assess T cell memory, activation, and effector status. (**B**) T cell exhaustion panel which included markers to assess regulatory T cells as well as CD8^+^ and CD4^+^ T cell markers of exhaustion. (PDF 4584 kb)

Additional file 13: **Figure S13:** Multiplex raw cellular densities. Raw cellular densities from multiplex analysis used for generation of Fig. 4b and c. For all samples *N* = 1 and *n* = 15 images per group (3 mice per group with 5 images assessed per tumor). (PDF 1479 kb)

Additional file 14: **Table S1.** Flow cytometry immune microenvironment staining panels. (DOCX 19 kb)

## Data Availability

The majority of data obtained and materials used are presented in this publication or in supplementary material. Additional data or materials will be provided upon reasonable request and signing of a material transfer agreement.

## Abbreviations

APCs: Antigen-presenting cells
CPR: CTX/L-NIL+ αPD-1/αCTLA-4 + radiation combination treatment
CTLA-4: Cytotoxic T lymphocyte associated antigen-4
CTX: Cyclophosphamide
DCs: Dendritic cells
HNSCC: Head and neck squamous cell carcinoma
HPV: Human papillomavirus
ICIs: Immune checkpoint inhibitors
iNOS: Inducible nitric oxide synthase
KLRG1: Killer cell lectin-like receptor
L-NIL: L-n6-(1-iminoethyl)-lysine
MDSC: Myeloid-derived suppressor cells
MHC: Major histocompatibility complex
PBMCs: Peripheral blood mononuclear cells
PD-1: Programmed cell death protein-1
pDCs: Plasmacytoid dendritic cells
RT: Radiotherapy
SBRT: Stereotactic body radiotherapy
STING: Stimulator of interferon genes
tdLN: Tumor-draining lymph node
TIME: Tumor immune microenvironment
Tregs: Regulatory T cells
t-SNE: t-stochastic neighbor embedding
