# Supplementary figures and images for: Immune microenvironment modulation unmasks therapeutic benefit of radiotherapy and checkpoint inhibition
Source: J Immunother Cancer. 2019 Aug 13;7:216. doi: 10.1186/s40425-019-0698-6 (PMC6693252; doi:10.1186/s40425-019-0698-6)

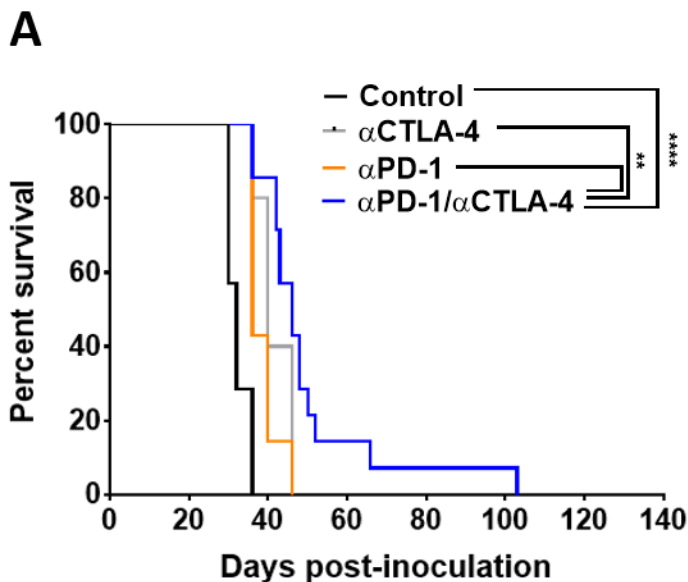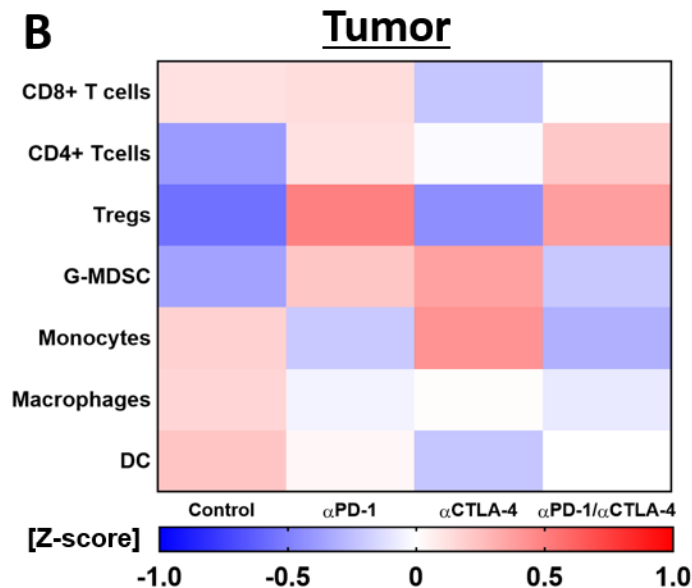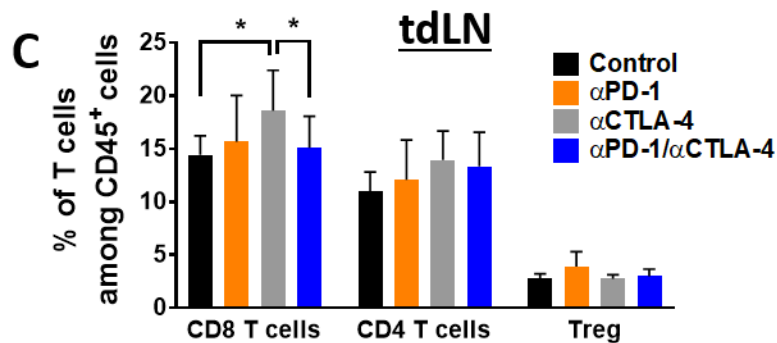

Supplement: Supplementary file 1 — Figure S1. Immune checkpoint inhibitors alone provide minimal treatment benefit or microenvironment improvement. (A-C) Subcutaneous established mEER tumors (day 17–18 post tumor cell injection) were treated with 6 total doses of αPD-1 (250 μg/dose) and/or αCTLA-4 (100 μg/dose) according to the schedule in (Fig. 1b), mice were euthanized when tumors reached 225 mm2. (A) Kaplan Meier survival curves with comparison between treatment groups (Log-rank test; N = 1 representative of 2; n = 5–12). (B) Flow cytometry immune profiling of treated tumors at 1 week of treatment (day 23) shown as z-scores for major lymphoid and myeloid subtypes between groups (N = 2; n = 9–12 per group). (C) Percentage of major lymphoid subsets (among CD45+ cells) in tdLN at day 23 of treatment for various treatment groups (Tukey’s multiple comparison test; N = 2; n = 9–12 per group). *p < 0.05; **p < 0.01; ***p < 0.001; ****p < 0.0001. (PDF 2648 kb) [file 40425_2019_698_MOESM1_ESM.pdf]

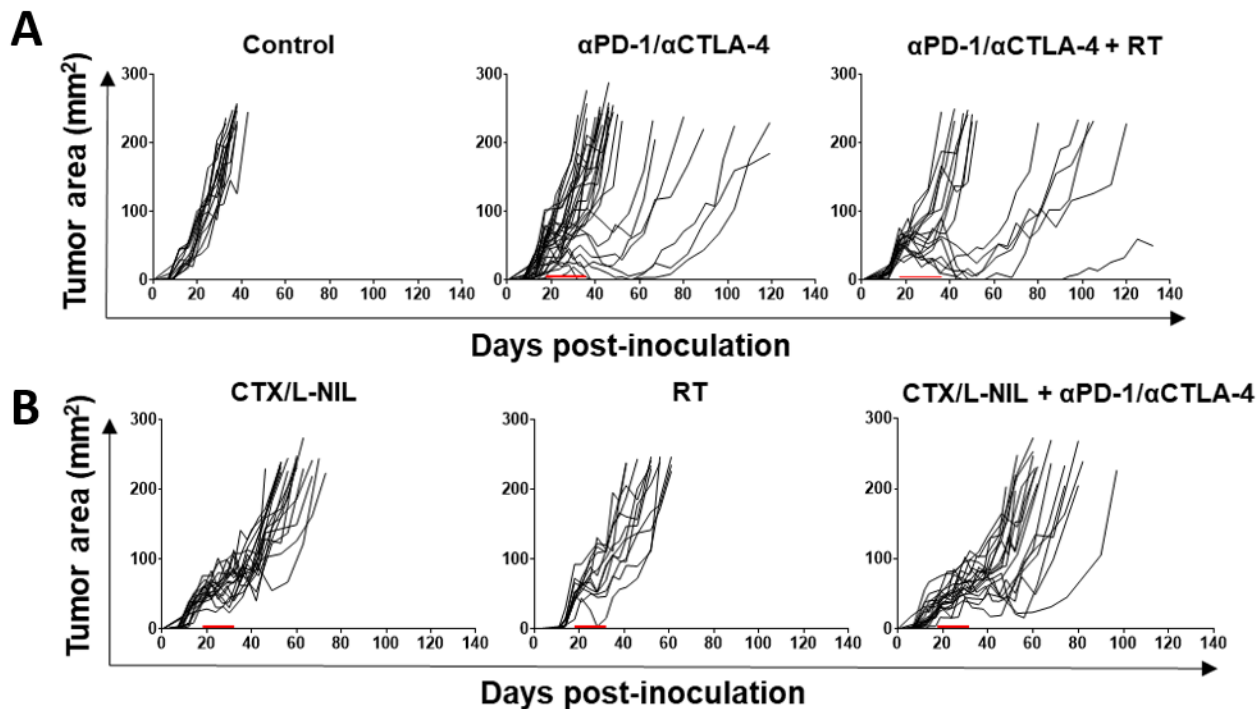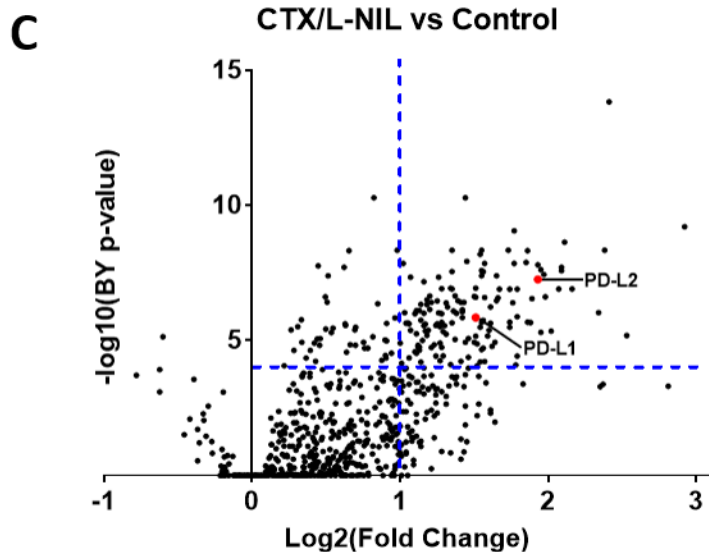

Supplement: Supplementary file 2 — Figure S2. Individual tumor growth curves for singlet and dual treatments and CTX/L-NIL gene expression. Subcutaneous established mEER tumors (day 17–18 post tumor cell injection) were treated with individual or dual treatment combinations of PD-1/CTLA-4, CTX/L-NIL, and radiation (RT) according to the same schedule shown in Figs. 1c and 2b. (A) Individual mEER tumor growth curves for 2 experiments, one of which was used for in Fig. 1d (N = 2; n = 7–17 per group). (B) Individual tumor growth curves for singlet and dual treatment combinations of CPR regimen (N = 2–3; n = 12–19). (C) Differential gene expression of CTX/L-NIL treated tumors compared to control tumors compared after 1 week (day 23) of treatment with PD-L1 and PD-L2 noted in red dots (N = 1; n = 9 per group). Blue lines indicate gene 2-fold change point (vertical) and corrected p-value less than 0.0001 (horizontal). (PDF 3329 kb) [file 40425_2019_698_MOESM2_ESM.pdf]

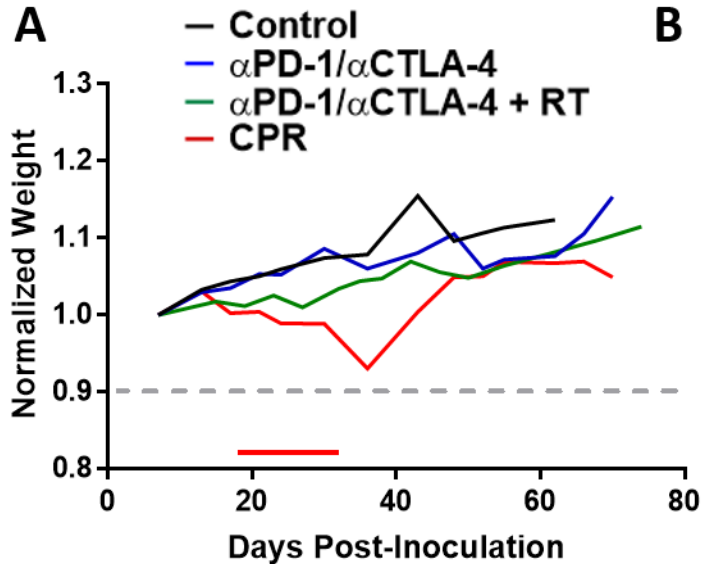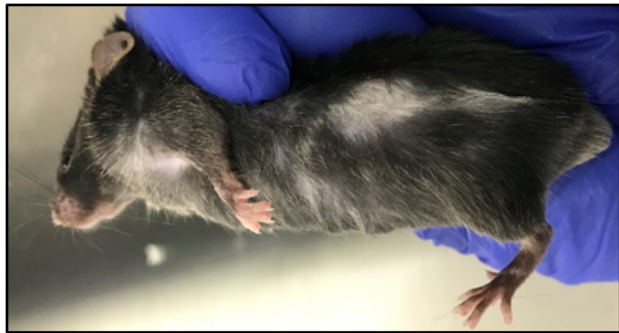

Supplement: Supplementary file 3 — Figure S3. CPR regimen induces minimal weight loss and no gross treatment related toxicities. (A) Normalized weight for treated mice over the course of treatment, normalized to mouse weight 1 week after tumor cell inoculation (N = 1 representative of 2; n = 5–9). (B) Image of mouse treated with full CPR regimen approximately 100 days after tumor clearance with white fur visible in location of tumor clearance. (PDF 1600 kb) [file 40425_2019_698_MOESM3_ESM.pdf]

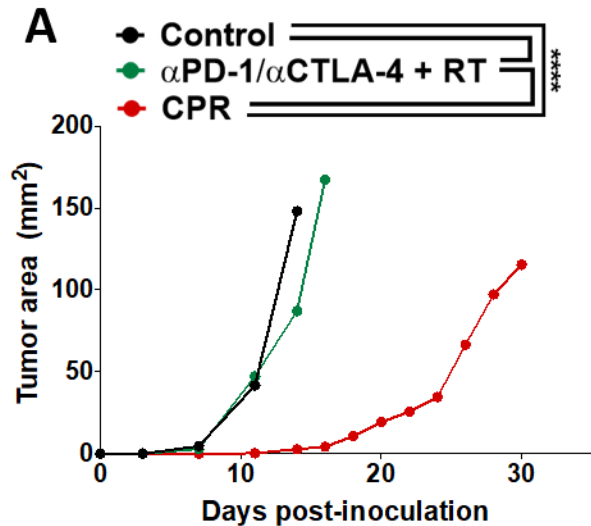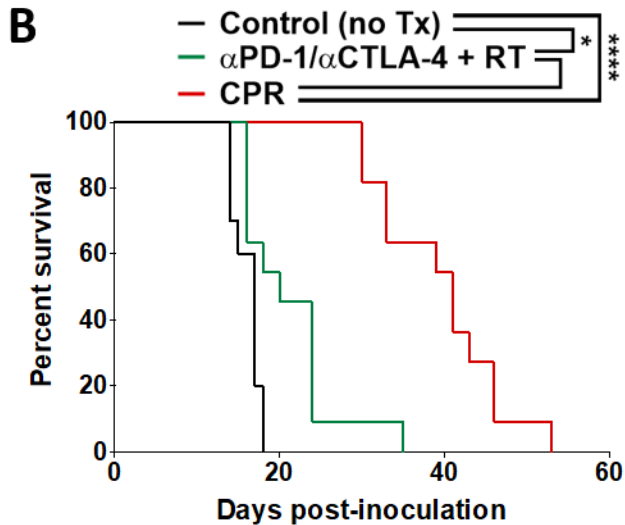

Supplement: Supplementary file 4 — Figure S4. CTX/L-NIL improves anti-tumor effect of αPD-1/αCTLA-4 and radiation in the B16 syngeneic melanoma tumor model. Subcutaneous established B16-F0 melanoma tumors (day 4 post tumor cell injection) were treated with αPD-1/αCTLA-4 and radiation alone, or combined with CTX/L-NIL immunomodulation (CPR regimen), mice were euthanized when tumors reached 225 mm2. (A) Average tumor area statistically compared at time of first control mouse euthanization (Tukey’s multiple comparison test; N = 1 representative of 2; n = 7–8 per group). (B) Kaplan Meier survival curves with comparison between treatment groups (Log-rank test; N = 2; n = 10–11 per group). *p < 0.05; ****p < 0.0001. (PDF 1425 kb) [file 40425_2019_698_MOESM4_ESM.pdf]

iNOS

Control

$\alpha$ PD-1/ $\alpha$ CTLA-4

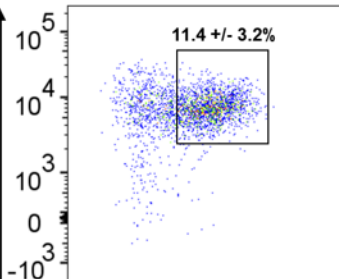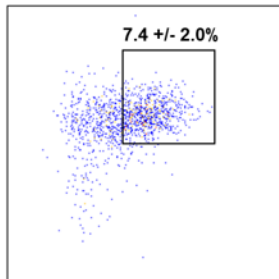

$\alpha$ PD-1/ $\alpha$ CTLA-4 + RT

CPR

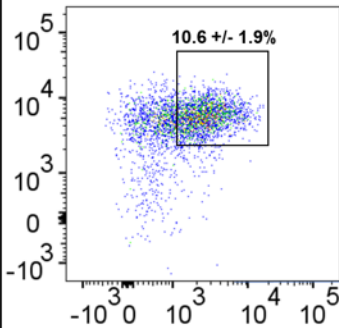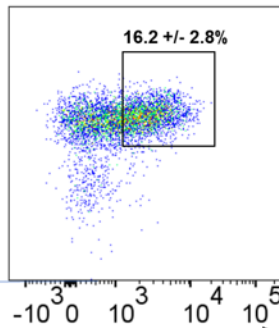

MHCII

Supplement: Supplementary file 5 — Figure S5. CPR increases intratumoral M1-like macrophages. Aggregate flow cytometry scatterplots showing MHCII and iNOS expression among tumor-dwelling macrophages at day 23 of treatment (percentages show mean +/− SD; N = 1 representative of 2; n = 4 aggregate samples per group). (PDF 1299 kb) [file 40425_2019_698_MOESM5_ESM.pdf]

## Tumor

# A

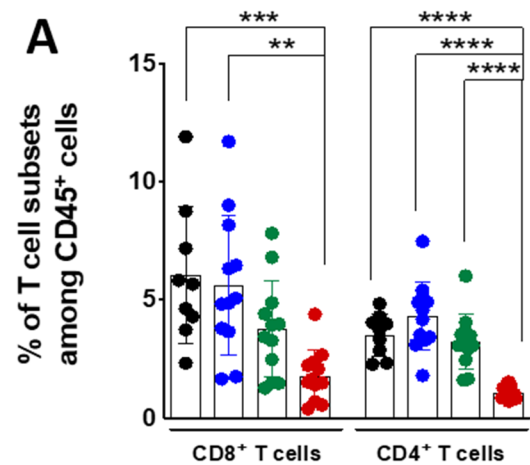

# B

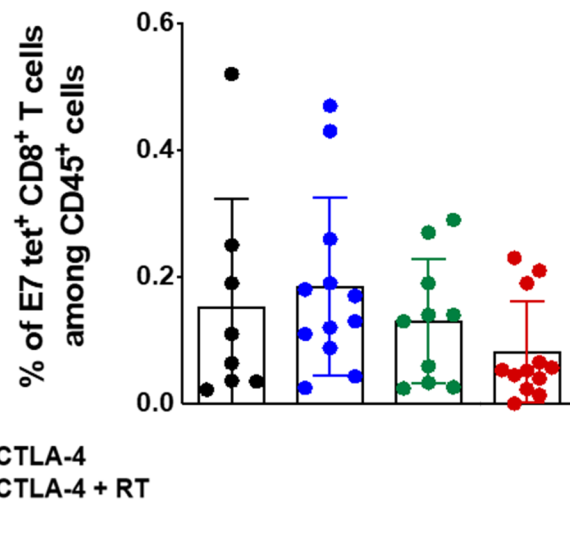

**C**

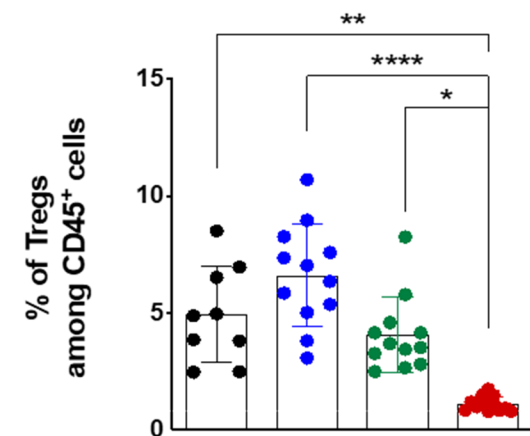

D

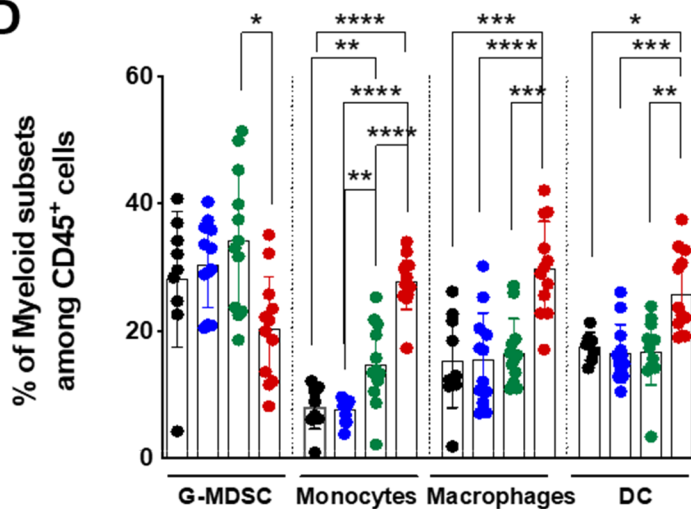

**E**

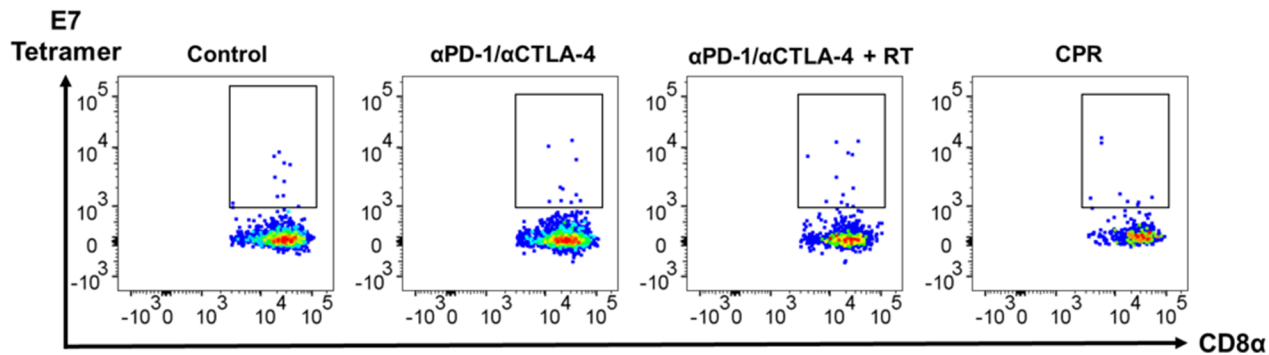

Supplement: Supplementary file 6 — Figure S6. Tumor immune microenvironment data at day 23. Flow cytometry assessment of tumor was performed at day 23 for all treatment groups and major immune cell subset percentages (among CD45+ cells) are shown. (A) Percentage of CD4+ and CD8+ T cell subsets. (B) Percentage E7 tetramer+ CD8+ T cells. (C) Percentage of Tregs. (D) Percentage of major myeloid subsets. (For A-D, Tukey’s multiple comparison test; N = 2; 8–13 per group). (E) Aggregate flow cytometry scatter plots of CD8+ T cells showing E7 tetramer staining (N = 1, representative of 2; n = 4 aggregate samples per group). *p < 0.05; **p < 0.01; ***p < 0.001; ****p < 0.0001. (PDF 15352 kb) [file 40425_2019_698_MOESM6_ESM.pdf]

# Tumor

**A**

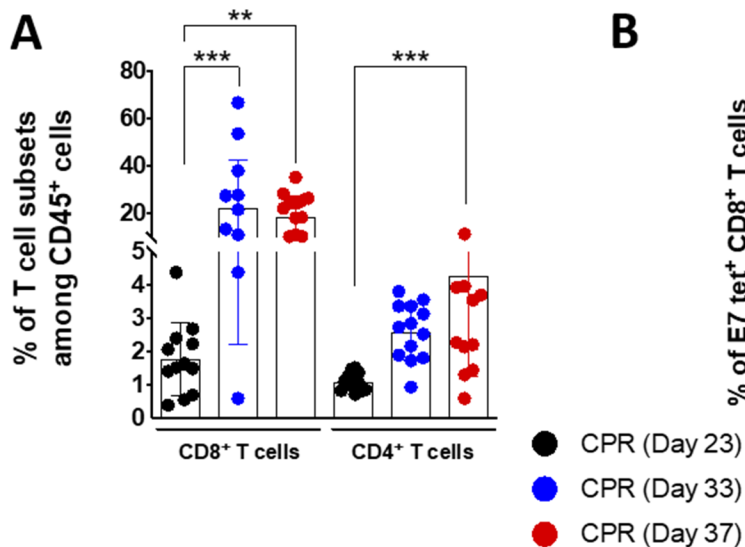

**B**

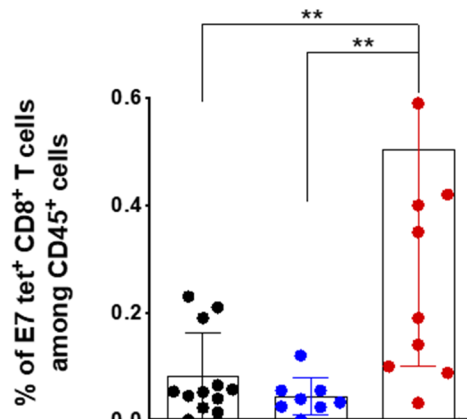

**C**

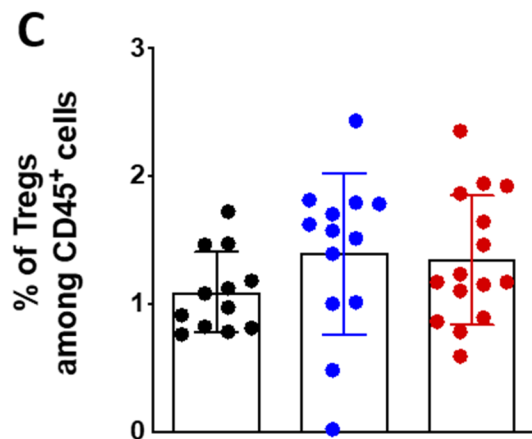

**D**

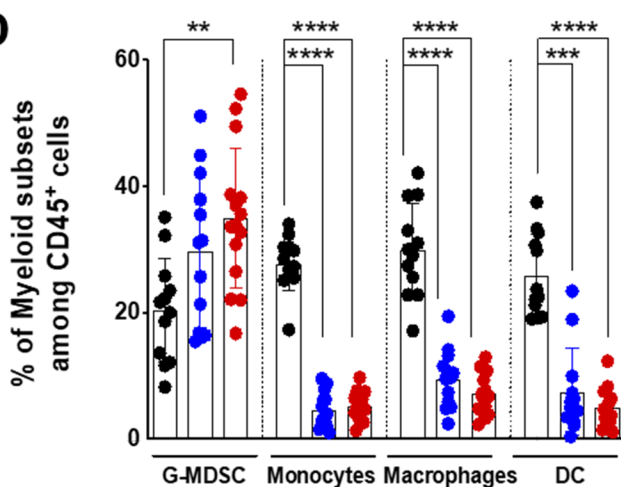

**E**

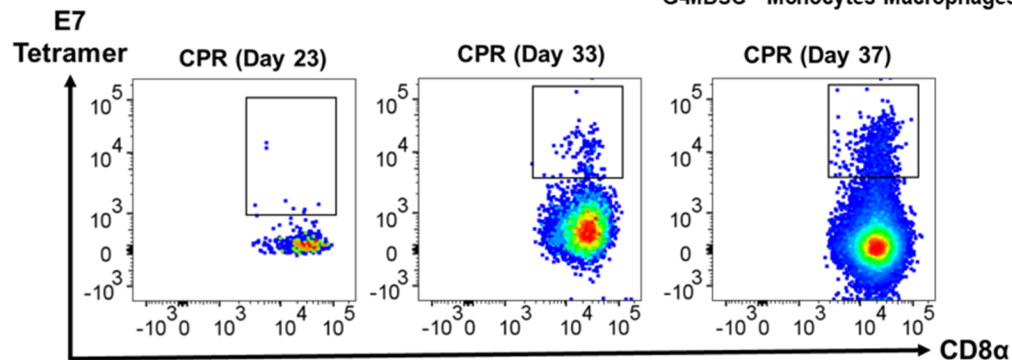

Supplement: Supplementary file 7 — Figure S7. Tumor immune microenvironment data time course. Flow cytometry assessment of tumor was performed at day 23, day 33, and day 37 for the CPR treatment group and major immune cell subset percentages (among CD45+ cells) are shown. (A) Percentage of CD4+ and CD8+ T cell subsets. (B) Percentage E7 tetramer+ CD8+ T cells. (C) Percentage of Tregs. (D) Percentage of major myeloid subsets. (For A-D, Tukey’s multiple comparison test; N = 2; 8–13 per group). (E) Aggregate flow cytometry scatter plots of CD8+ T cells showing E7 tetramer staining (N = 1, representative of 2; n = 4 aggregate samples per group). **p < 0.01; ***p < 0.001; ****p < 0.0001. (PDF 14226 kb) [file 40425_2019_698_MOESM7_ESM.pdf]

# dLNs

**A**

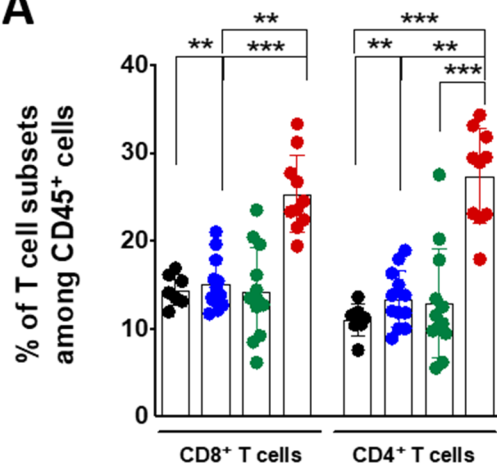

**B**

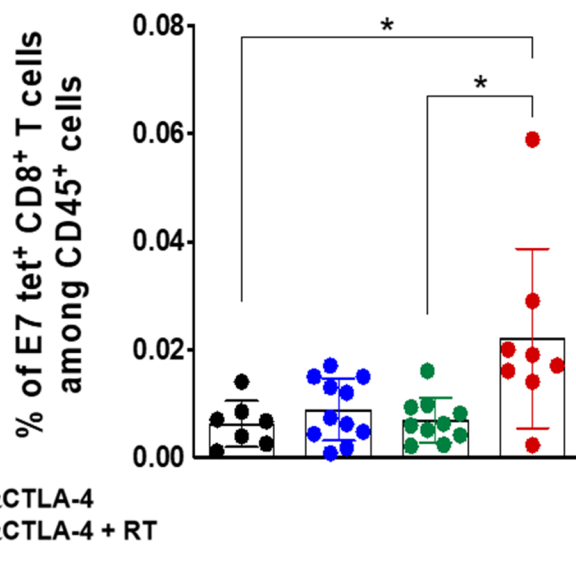

**C**

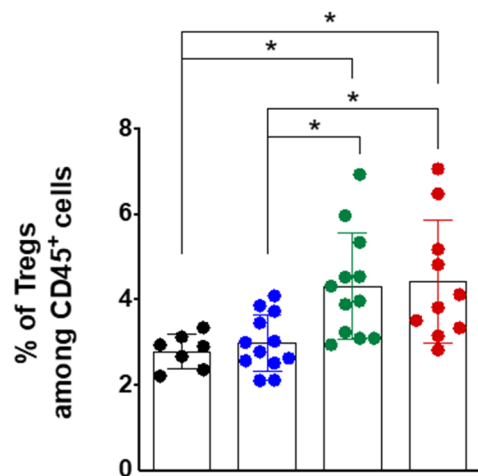

**D**

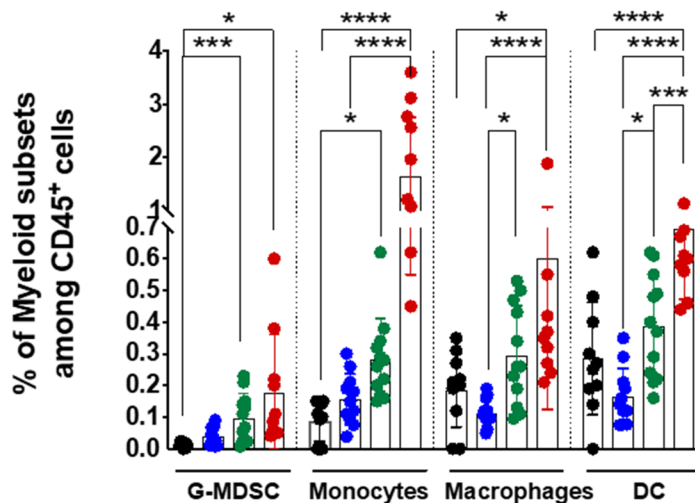

**E**

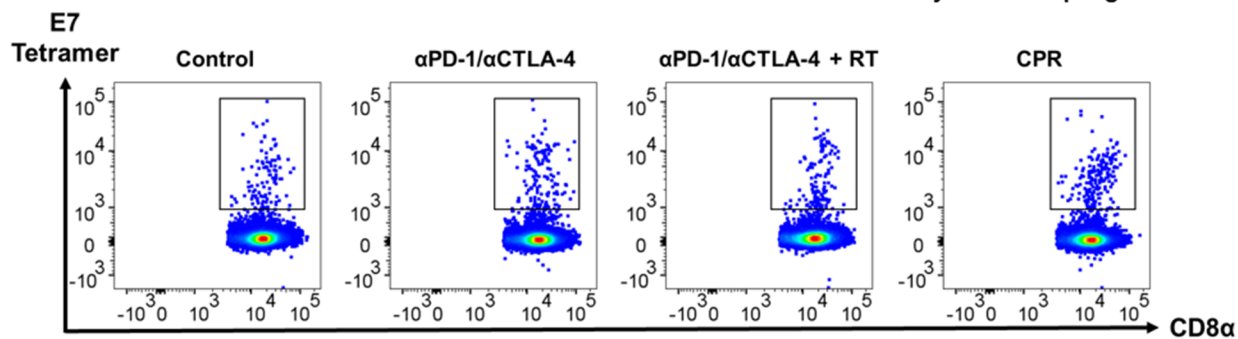

Supplement: Supplementary file 8 — Figure S8. tdLN immune microenvironment data at day 23. Flow cytometry assessment of tdLN was performed at day 23 for all treatment groups and major immune cell subset percentages (among CD45+ cells) are shown. (A) Percentage of CD4+ and CD8+ T cell subsets. (B) Percentage E7 tetramer+ CD8+ T cells. (C) Percentage of Tregs. (D) Percentage of major myeloid subsets. (For A-D, Tukey’s multiple comparison test; N = 2; 7–13 per group). (E) Aggregate flow cytometry scatter plots of CD8+ T cells showing E7 tetramer staining (N = 1, representative of 2; n = 4 aggregate samples per group). *p < 0.05; **p < 0.01; ***p < 0.001; ****p < 0.0001. (PDF 15328 kb) [file 40425_2019_698_MOESM8_ESM.pdf]

# dLNs

**A**

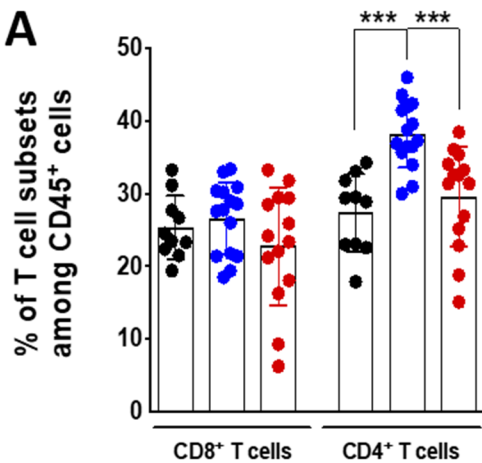

**B**

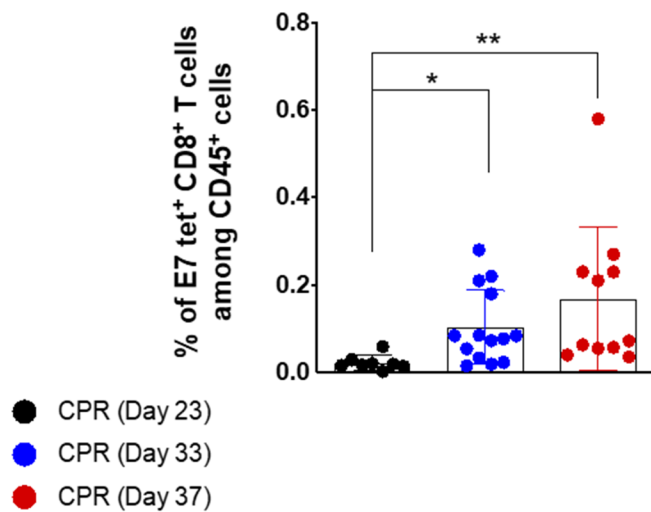

**C**

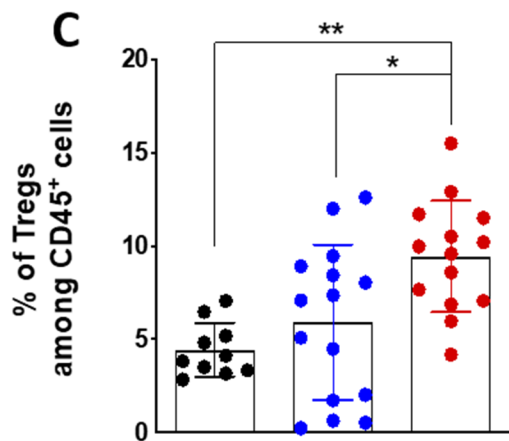

**D**

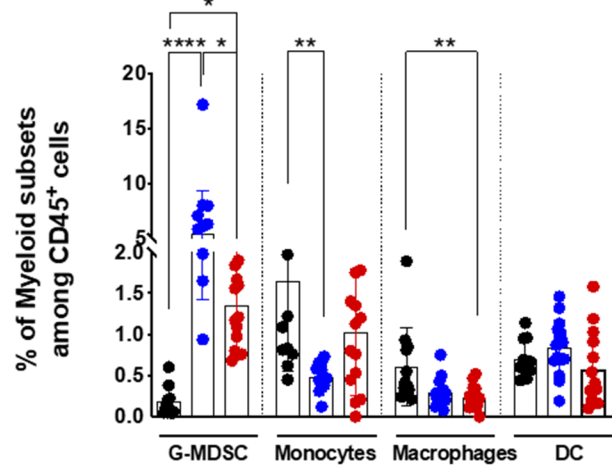

**E**

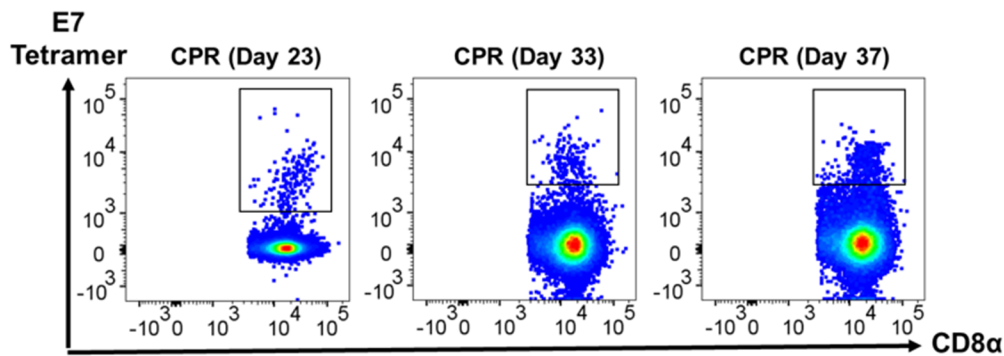

Supplement: Supplementary file 9 — Figure S9. tdLN immune microenvironment data time course. Flow cytometry assessment of tdLN was performed at day 23, day 33, and day 37 for the CPR treatment group and major immune cell subset percentages (among CD45+ cells) are shown. (A) Percentage of CD4+ and CD8+ T cell subsets. (B) Percentage E7 tetramer+ CD8+ T cells. (C) Percentage of Tregs. (D) Percentage of major myeloid subsets. (For A-D, Tukey’s multiple comparison test; N = 2; 7–13 per group). (E) Aggregate flow cytometry scatter plots of CD8+ T cells showing E7 tetramer staining (N = 1, representative of 2; n = 4 aggregate samples per group). *p < 0.05; **p < 0.01; ***p < 0.001; ****p < 0.0001. (PDF 14769 kb) [file 40425_2019_698_MOESM9_ESM.pdf]

**A****Tumor**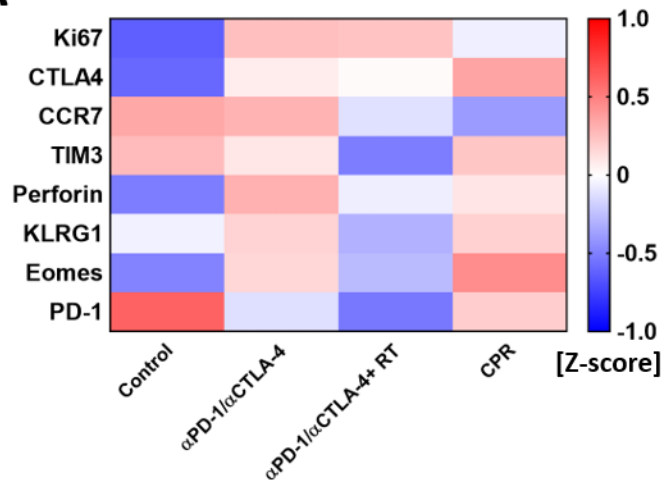**B****tdLN**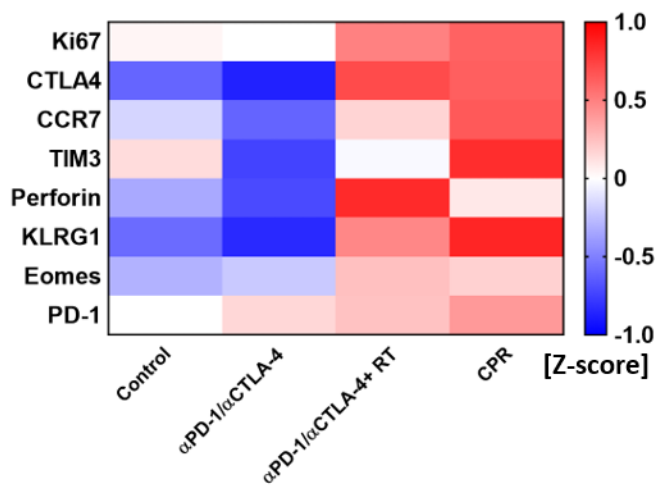**C****tdLN**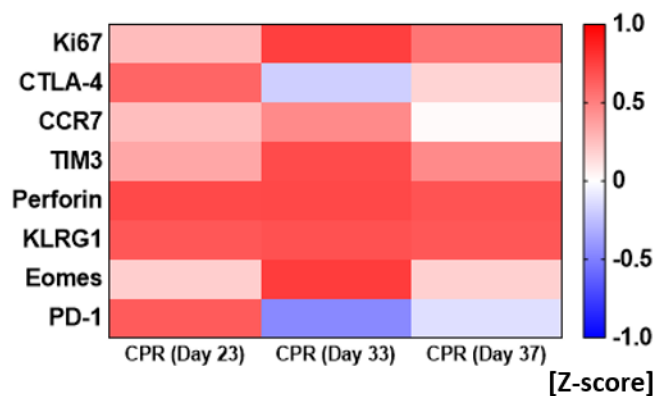

Supplement: Supplementary file 10 — Figure S10. CD8+ T cell phenotype microenvironment heatmaps. Flow cytometry assessment of CD8+ T cell phenotype changes at day 23 for all treatment groups, and further assessed at day 33 and 37 for CPR treatment and compared to tumor size-matched control mice. Data is represented as a z-score of the phenotypic marker’s median fluorescence intensity (MFI) among CD8+ T cells represented as z-scores. (A) Tumor and (B) tdLN CD8+ T cell phenotypic marker expression between all treatment groups at day 23 of treatment (N = 2, n = 7–13 per group). (C) tdLN CD8+ T cell phenotypic marker expression at days 23, 33, and 37 of CPR treatment progression compared to size-matched control tumors (N = 2; n = 7–13 per group). (PDF 3118 kb) [file 40425_2019_698_MOESM10_ESM.pdf]

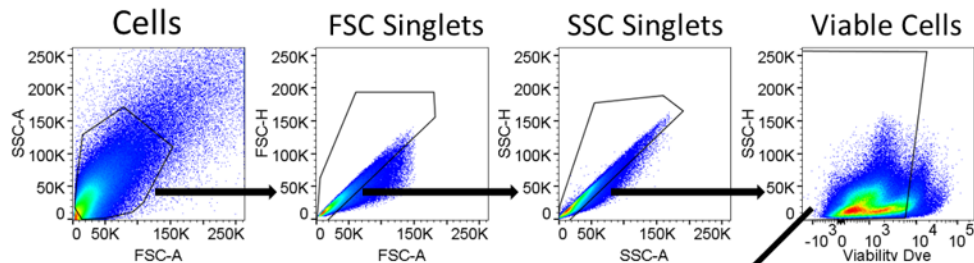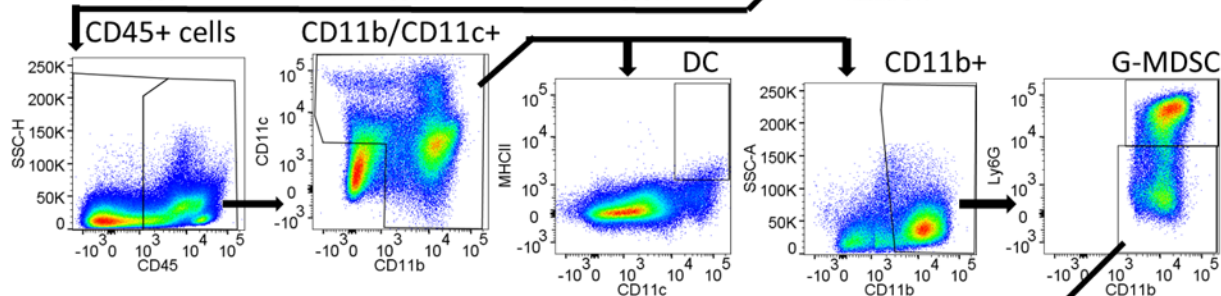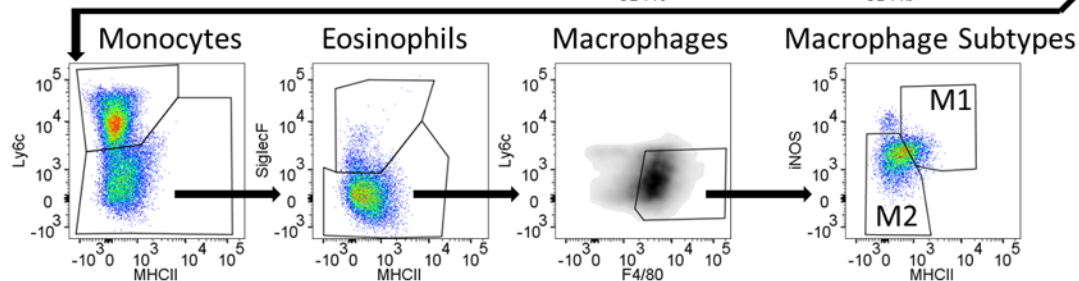

Supplement: Supplementary file 11 — Figure S11. Myeloid flow cytometry gating strategy. Flow cytometry strategy used for gating myeloid subsets, with labeled cell type above the respective gate. (PDF 2002 kb) [file 40425_2019_698_MOESM11_ESM.pdf]

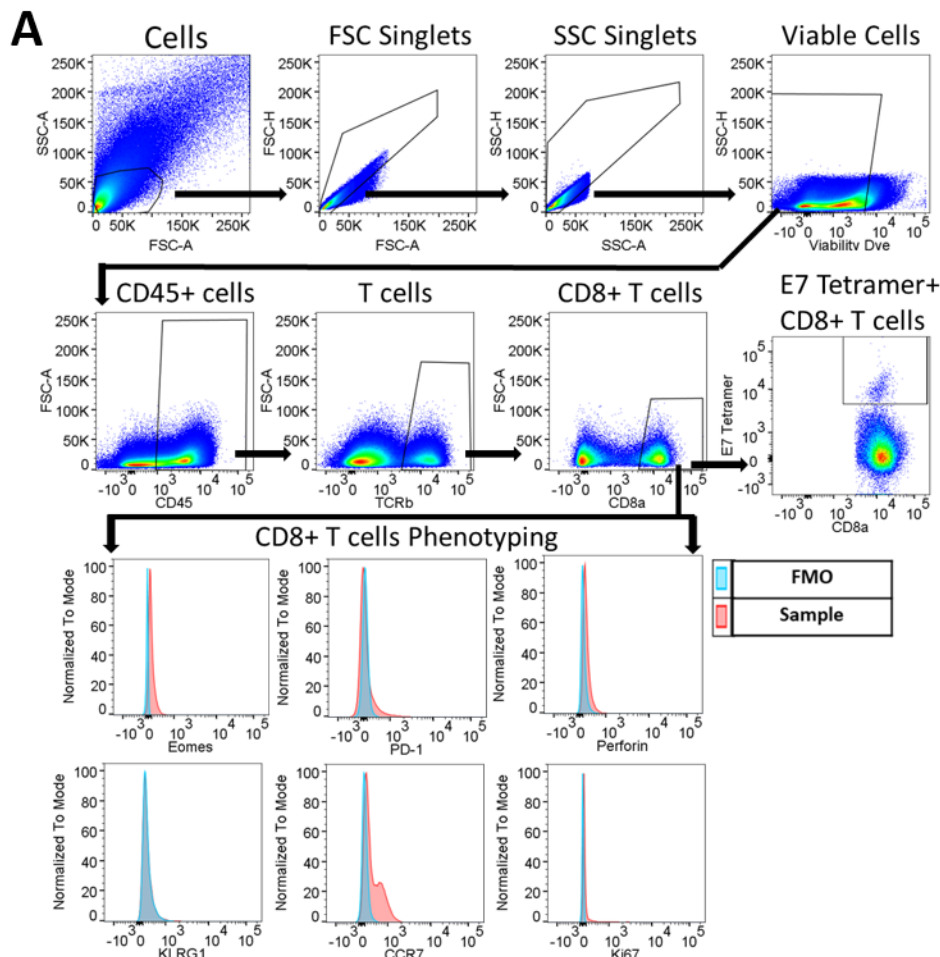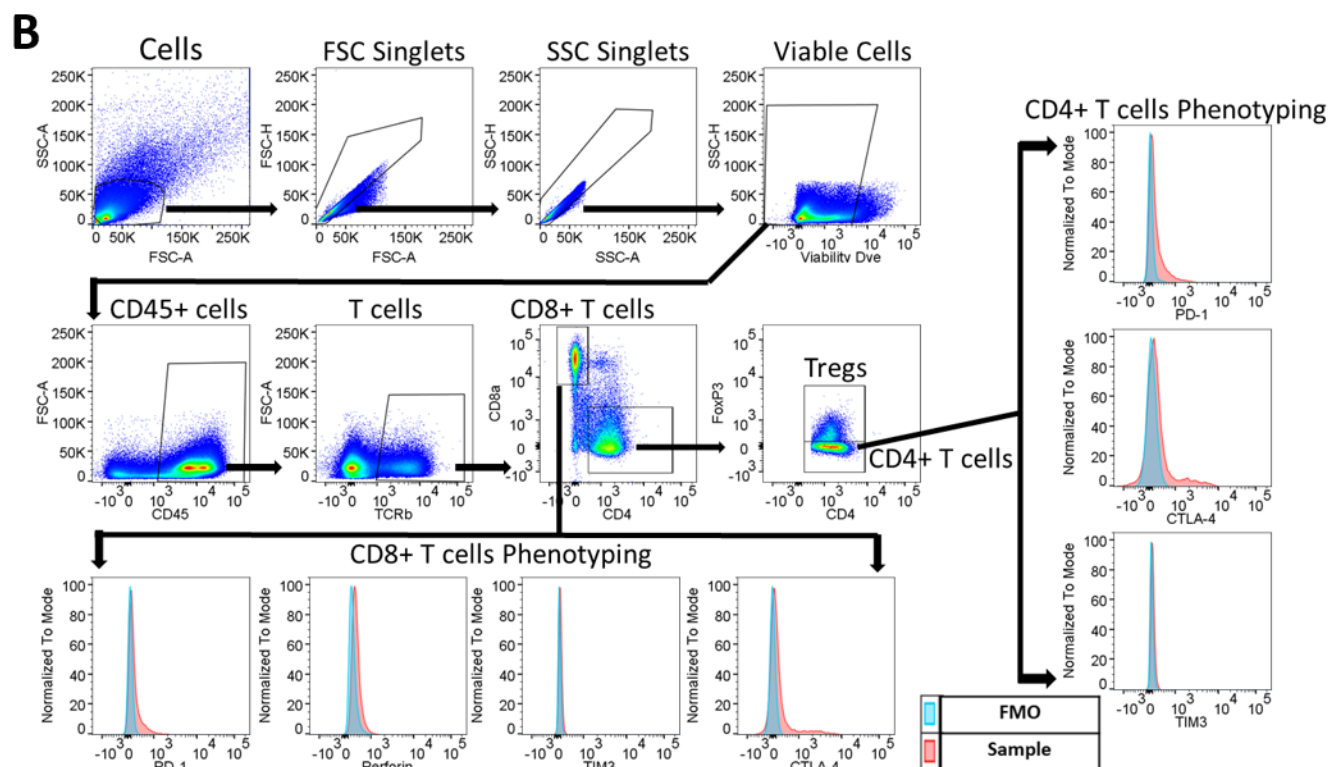

Supplement: Supplementary file 12 — Figure S12. Lymphoid flow cytometry gating strategies. Flow cytometry strategy used for gating lymphoid subsets, with labeled cell type above the respective gate. (A) T cell phenotype panel which included various markers to assess T cell memory, activation, and effector status. (B) T cell exhaustion panel which included markers to assess regulatory T cells as well as CD8+ and CD4+ T cell markers of exhaustion. (PDF 4584 kb) [file 40425_2019_698_MOESM12_ESM.pdf]

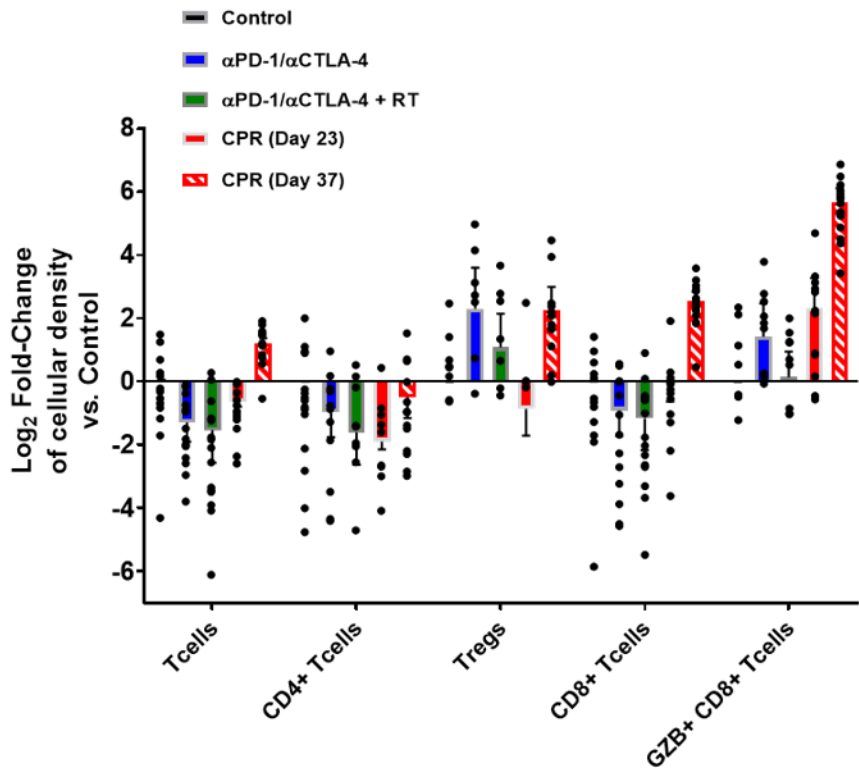

Supplement: Supplementary file 13 — Figure S13: Multiplex raw cellular densities. Raw cellular densities from multiplex analysis used for generation of Fig. 4b and c. For all samples N = 1 and n = 15 images per group (3 mice per group with 5 images assessed per tumor). (PDF 1479 kb) [file 40425_2019_698_MOESM13_ESM.pdf]
