# Supplementary material for: Immune microenvironment modulation unmasks therapeutic benefit of radiotherapy and checkpoint inhibition
Source: J Immunother Cancer. 2019 Aug 13;7:216. doi: 10.1186/s40425-019-0698-6 (PMC6693252; doi:10.1186/s40425-019-0698-6)
Supplement: Supplementary file 14 — Table S1. Flow cytometry immune microenvironment staining panels. (DOCX 19 kb) [file 40425_2019_698_MOESM14_ESM.docx]

**Table S1. Flow cytometry immune microenvironment staining panels.**

| **Marker** | **Fluorophore** | **Clone** | **Extracellular (E) or Intracellular (I)** | **Dilution** | **Source** |
| --- | --- | --- | --- | --- | --- |
| **Myeloid Panel** | |  |  |  |  |
| **LIVE/DEAD Fixable Blue** | DAPI | N/A | N/A | 1/500 | ThermoFisher |
| **α-CD45** | APC-efluor780 | 30-F11 | E | 1/250 | Invitrogen |
| **α-MHCII** | AF700 | MS/114.15.2 | E | 1/800 | eBioscience |
| **α-CD11c** | PE-Cy5 | N418 | E | 1/400 | Invitrogen |
| **α-F4/80** | Pacific Blue | BM8 | E | 1/250 | eBioscience |
| **α-CD11b** | BV650 | M1/70 | E | 1/400 | BD Biosciences |
| **α-Ly6C** | PerCP-Cy5.5 | HK1.4 | E | 1/200 | Invitrogen |
| **α-Ly6G** | PE | RB6-8CS | E | 1/300 | eBioscience |
| **α-SiglecF** | BV711 | E50-2440 | E | 1/200 | BD Biosciences |
| **α-PD-L1** | PE-Cy7, | MIH5 | E | 1/200 | eBioscience |
| **α-iNOS** | FITC | 6/iNOS/NOS Type II | I | 1/50 | BD Biosciences |
| **T cell Phenotype Panel** | |  |  |  |  |
| **LIVE/DEAD Fixable Blue** | DAPI | N/A | N/A | 1/500 | ThermoFisher |
| **α-CD45** | APC-efluor780 | 30-F11 | E | 1/250 | Invitrogen |
| **α-TcRβ** | PE-Cy5 | HS7-597 | E | 1/800 | eBioscience |
| **α-CD8α** | PE, FITC | KT15 | E | 1/200 | Santa Cruz Biotechnology, Invitrogen |
| **α-PD-1** | PerCP-efluor710 | J43 | E | 1/200 | eBioscience |
| **α-CCR7** | BV605 | 4B12 | E | 1/50 | BD Biosciences |
| **α-KLRG1** | BV510 | 2F1 | E | 1/100 | BD Biosciences |
| **E7-tetramer** | BV421 | H-2D(b) RAHYNIVTF | E | 1/100 | NIH Tetramer Facility |
| **α-Perforin** | FITC, APC | eBioOMAK-D | I | 1/50 | Invitrogen |
| **α-Eomes** | PE-Cy7 | Dan11mag | I | 1/25 | Invitrogen |
| **α-Ki67** | AF700 | SoLA15 | I | 1/50 | Invitrogen |
| **T cell Exhaustion Panel** | |  |  |  |  |
| **LIVE/DEAD Fixable Blue** | DAPI | N/A | N/A | 1/500 | ThermoFisher |
| **α-CD45** | APC-efluor780 | 30-F11 | E | 1/250 | Invitrogen |
| **α-TcRβ** | PE-Cy5 | HS7-597 | E | 1/800 | eBioscience |
| **α-CD8α** | PE | KT15 | E | 1/200 | Santa Cruz Biotechnology |
| **α-CD4** | BUV737 | GK1.5 | E | 1/200 | BD Biosciences |
| **α-TIM3** | PE-Cy7, APC | RMT3-23, 8B.2C12 | E | 1/100 | Invitrogen, eBioscience |
| **α-PD-1** | PE-CF594, PerCP-efluor710 | J43 | E | 1/200 | BD Biosciences, eBioscience |
| **α-CTLA-4** | APC-R700 | UC10-4F10-11 | E | 1/100 | BD Biosciences |
| **α-Perforin** | APC, FITC | eBioOMAK-D | I | 1/50 | Invitrogen |
| **α-FOXP3** | FITC, PE-Cy7 | FJK-16s | I | 1/50 | Invitrogen |
| **Cancer Cell Panel** | |  |  |  |  |
| **LIVE/DEAD Fixable Blue** | DAPI | N/A | N/A | 1/500 | ThermoFisher |
| **α-CD45** | APC-efluor780 | 30-F11 | E | 1/250 | Invitrogen |
| **α-PD-L1** | PE-Cy7 | MIH5 | E | 1/200 | eBioscience |
| **α-PD-L2** | PerCP-eFluor710 | 122 | E | 1/200 | eBioscience |
| **α-MHCI (H-2Db)** | BV510 | KH95 | E | 1/200 | BD Biosciences |
| **α-iNOS** | APC | CXNFT | I | 1/50 | eBioscience |
| **α-Ki67** | BV711 | B56 | I | 1/100 | BD Bioscience |
